# Supplementary figures and images for: Spatacsin regulates directionality of lysosome trafficking by promoting the degradation of its partner AP5Z1
Source: PLoS Biol. 2023 Oct 23;21(10):e3002337. doi: 10.1371/journal.pbio.3002337 (PMC10621996; doi:10.1371/journal.pbio.3002337)

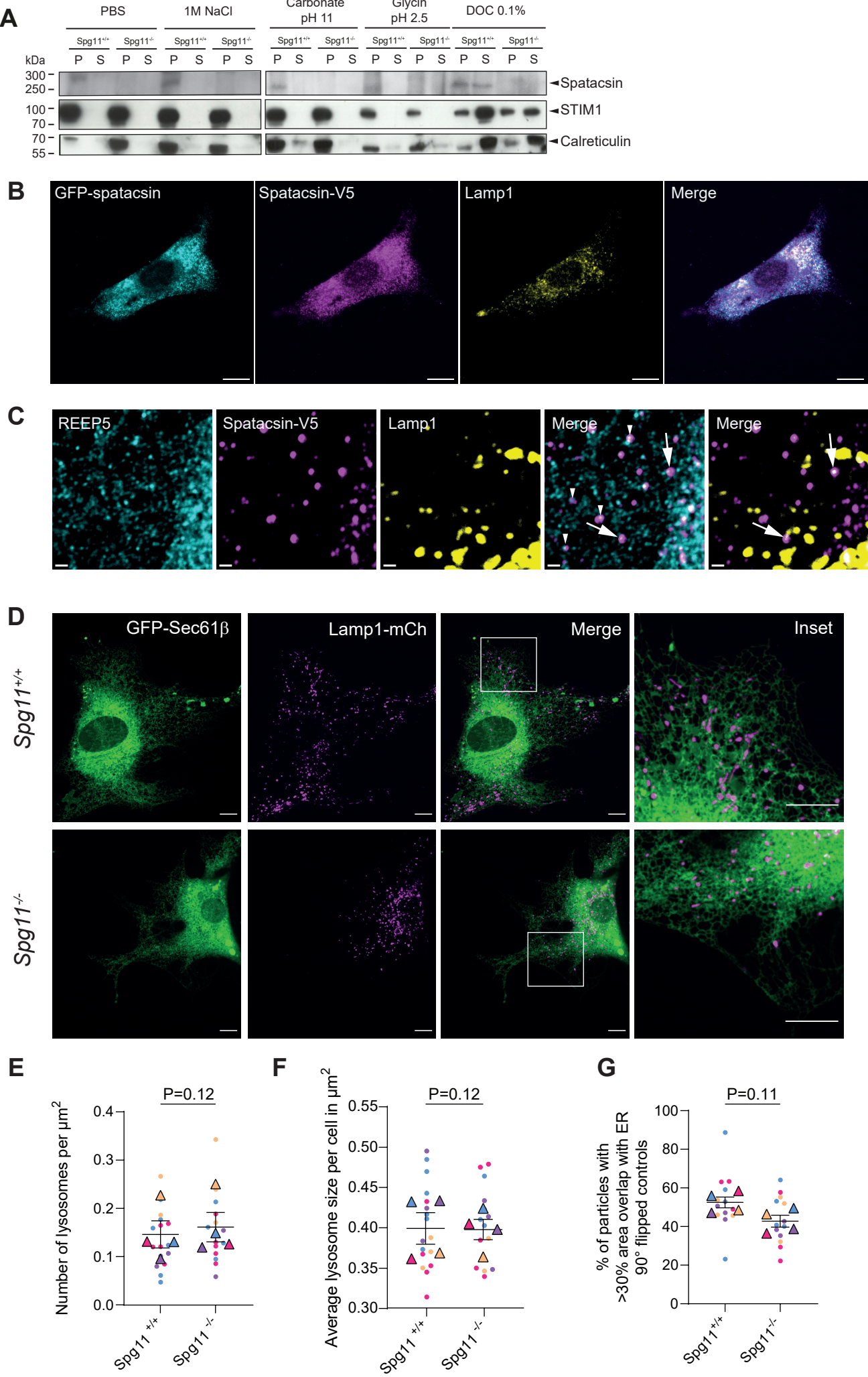

Supplement: S1 Fig — (A). Membrane fractions of Spg11+/+ and Spg11−/− mouse brains were resuspended in the indicated buffers or detergents and refractionated into the supernatant (S) and membrane pellet (P). Spatacsin was released from membranes only with the detergent deoxycholate (DOC), like the transmembrane protein STIM1. (B). MEFs expressing spatacsin constructs with an N-terminal GFP tag or a C-terminal V5-tag. Cells were immunostained with anti-V5 antibody, anti-GFP antibody, and the lysosome marker Lamp1. Scale bar: 5 μm. (C) STED images of MEFs expressing V5-tagged spatacsin. Cells were immunostained with anti-V5, anti-endogenous ER protein REEP5, and anti-LAMP1 antibodies. Scale bar: 1 μm. Arrowheads point spatacsin-V5 colocalized with the ER marker REEP5. Spatacsin V5 occasionnaly colocalized with the lysosome marker Lamp1 and the ER marker REEP5 (arrows). (D) Live imaging of the ER marker GFP-Sec61β and lysosome marker Lamp1-mCherry in Spg11+/+ and Spg11−/− MEFs. Note that the absence of spatacsin (Spg11−/−) did not alter ER morphology. Scale bar: 5 μm. (E) Quantification of the average number of lysosomes per square micrometer in Spg11+/+ and Spg11−/− MEFs. Superplot: means and SEM, N = 14 cells from 4 independent experiments. Paired t test on the means. (F) Quantification of the average lysosomal size per cell in Spg11+/+ and Spg11−/− MEFs. Superplot: means and SEM, N = 14 cells from 4 independent experiments. Paired t test on the means. (G) Quantification of the proportion of lysosomes that have an area overlapping with the ER > 30% in Spg11+/+ and Spg11−/− MEFs when lysosomal staining was flipped by 90°. Superplot: means and SEM, N = 14 cells from 4 independent experiments. Paired t test on the means. Note that the values are much lower than the ones in Fig 1I, showing that the overlap observed in Fig 1I is not due to random colocalization. The raw data underlying panels E, F, and G can be found in S1 Data file. (PDF) [file pbio.3002337.s001.pdf]

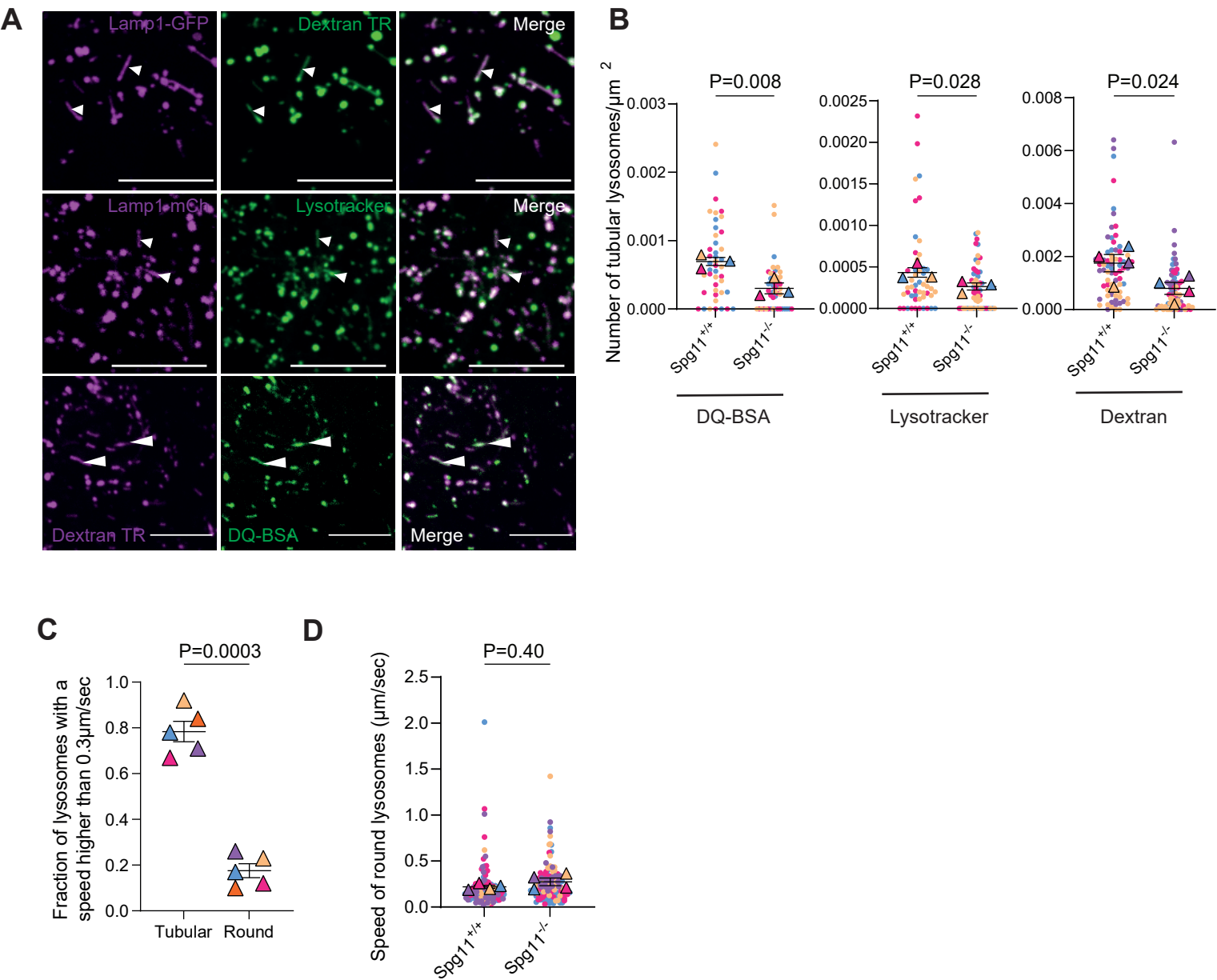

Supplement: S2 Fig — (A). Live imaging of lysosomes stained with various markers in Spg11+/+ MEFs. Note that tubular lysosomes (white arrows) were positive for Lamp1, 10 kDa Dextran-Texas Red (TR), Lysotracker green, as well as DQ-BSA green, indicating that they are acidic and catalytically active compartments. Scale bar: 5 μm. (B) Quantification of the number of tubular lysosomes in Spg11+/+ and Spg11−/− MEFs using the fluorescent markers DQ-BSA, Lysotracker, or Dextran. Superplot: means and SEM, N > 40 cells from 3 independent experiments. Paired t tests on the means. (C) Quantification of the proportion of lysosomes with an average speed > 0.3 μm/s according to their shape in wild-type MEFs. Mean and SEM, paired t test. (D) Quantification of the average speed of round lysosomes in Spg11+/+ and Spg11−/− MEFs. Superplot: means and SEM, N > 87 cells from 4 independent experiments. Paired t test on the means. The raw data underlying panels B, C, and D can be found in S1 Data file. (PDF) [file pbio.3002337.s002.pdf]

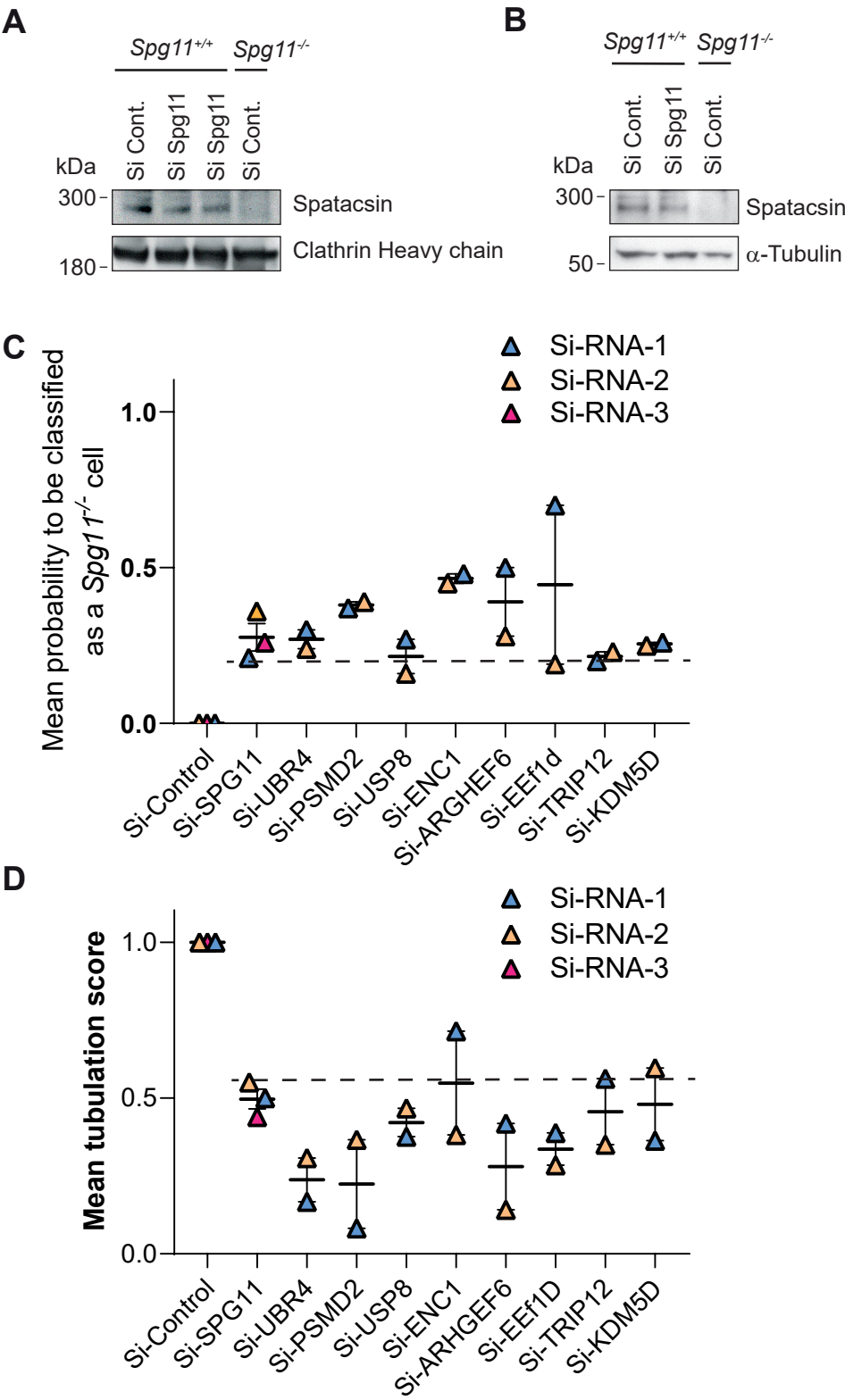

Supplement: S4 Fig — (A, B) Western blots of wild-type MEFs transfected with control siRNA or independent siRNA that down-regulate spatacsin purchased from Dharmacon (A) or ThermoFisher (B). Lysate of Spg11−/− MEFs was used as a negative control. Equal loading was validated by clathrin heavy chain (A) or α-tubulin (B) immunoblotting. (C) Graphs showing the mean probability of MEFs transfected with indicated siRNA to be considered as a knockout by the trained neural network, based on the analysis of the lysosomal staining with Dextran-Texas Red. Two independent siRNAs were tested for each target gene and 3 for Spg11. Dashed line corresponds to the minimal effect obtained with an siRNA down-regulating Spg11. (D) Graph showing the mean tubulation scores when candidates interactors of spatacsin were down-regulated by siRNA. Dashed line corresponds to the minimal effect obtained with an siRNA down-regulating Spg11. The raw data underlying panels C and D can be found in S1 Data file. (PDF) [file pbio.3002337.s004.pdf]

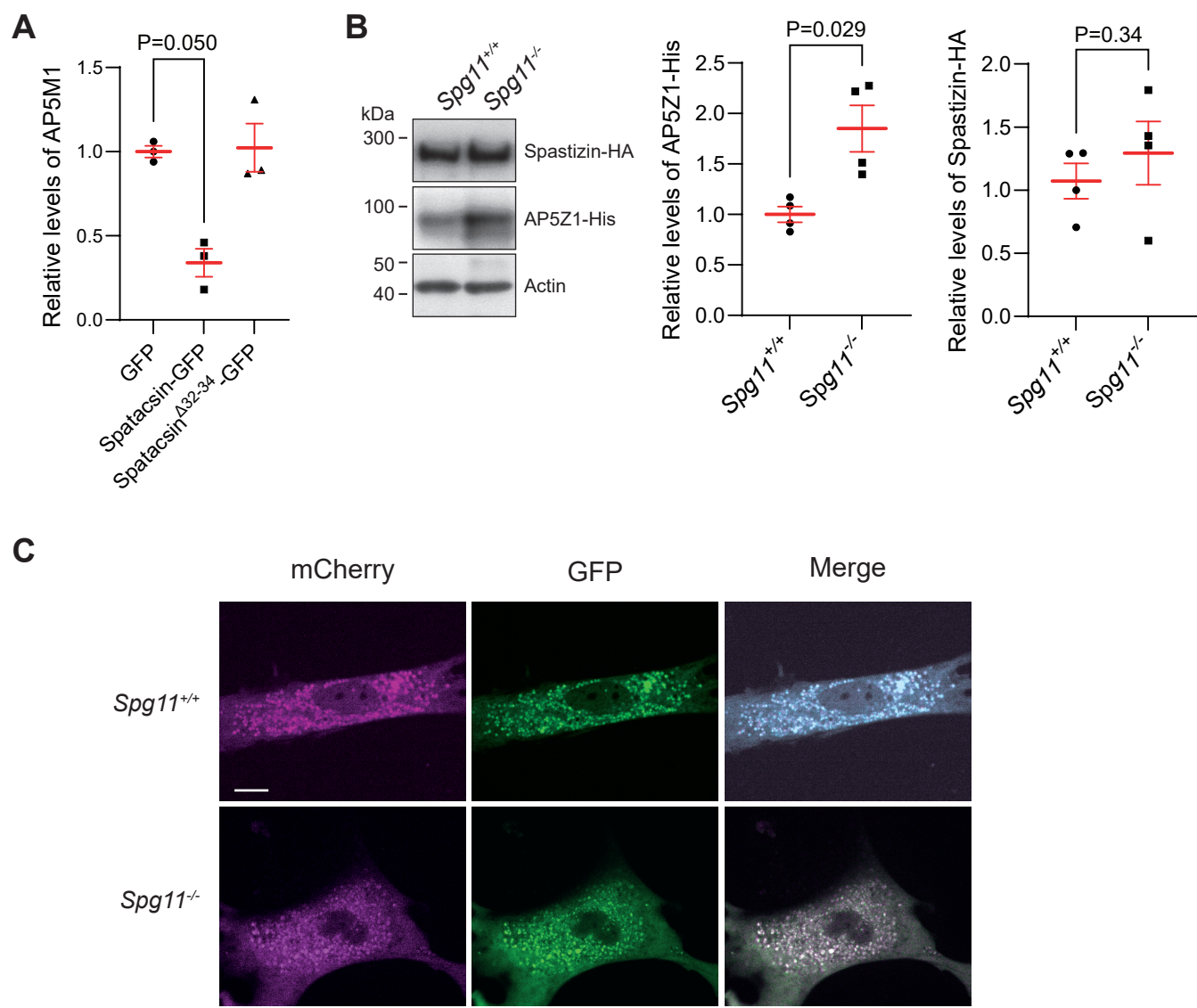

Supplement: S5 Fig — (A) Quantification of AP5M1 levels by western blot (Fig 4A): means and SEM, N = 3 independent experiments. Kruskal–Wallis test. (B) Western blot with anti-AP5Z1 antibody and anti-HA antibody upon transfection of Spg11+/+ and Spg11−/− MEFs with vector overexpressing AP5Z1-His and spastizin-HA. Right: quantification of relative levels of AP5Z1-His and Spastizin-HA. Means and SEM, N = 4 independent experiments. Mann–Withney test. (C) Live images of Spg11+/+ and Spg11−/− MEFs expressing GFP-mCherry-AP5Z1 treated with bafilomycin 100 nM for 16 hours. Note that GFP and mCherry signals perfectly colocalize. Scale bar: 10 μm. The raw data underlying panels A and B can be found in S1 Data file. (PDF) [file pbio.3002337.s005.pdf]

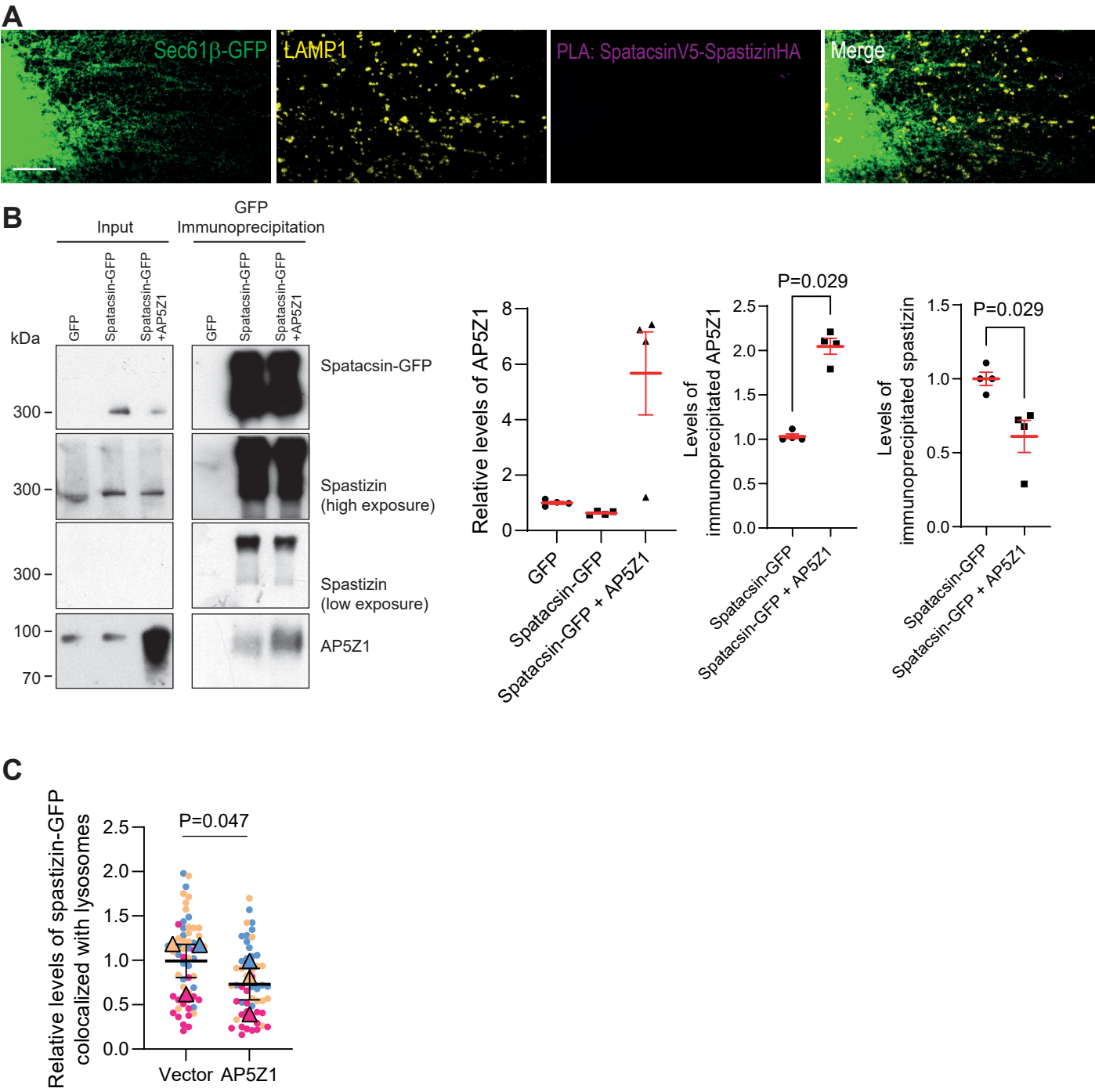

Supplement: S6 Fig — (A) Negative control for the proximity ligation assay (PLA) used to show the interaction between V5-tagged spatacsin and HA-tagged spastizin (Fig 5C). The PLA signal (magenta) is almost absent. Scale bar 10 μm. (B) Co-immunoprecipitation of spastizin and AP5Z1 with spatacsin-GFP upon overexpression of AP5Z1. Input represents 5% of lysate added to the immunoprecipitation assay. Inputs and immunoprecipitates were loaded on 2 separate gels processed simultaneously. Note that spatacsin and spastizin looked slightly different in input and co-immunoprecipitation, likely due to the high amount of both proteins in immunoprecipitates. Right: quantification of the amount of AP5Z1 in the input, as well as the relative amount of AP5Z1 or spastizin co-immunoprecipitated with Spatacsin-GFP. Means and SEM, N = 4 independent experiments. Mann–Whitney test. Note that the interaction of spatacsin-GFP with spastizin decreases when AP5Z1 is overexpressed. (C) Quantification of the proportion of spastizin-GFP colocalized with Lamp1-mCherry in wild-type MEFs overexpressing AP5Z1. Superplot: means and SEM, N > 53 cells from 3 independent experiments. Paired t test on the means. The raw data underlying panels B and C can be found in S1 Data file. (PDF) [file pbio.3002337.s006.pdf]

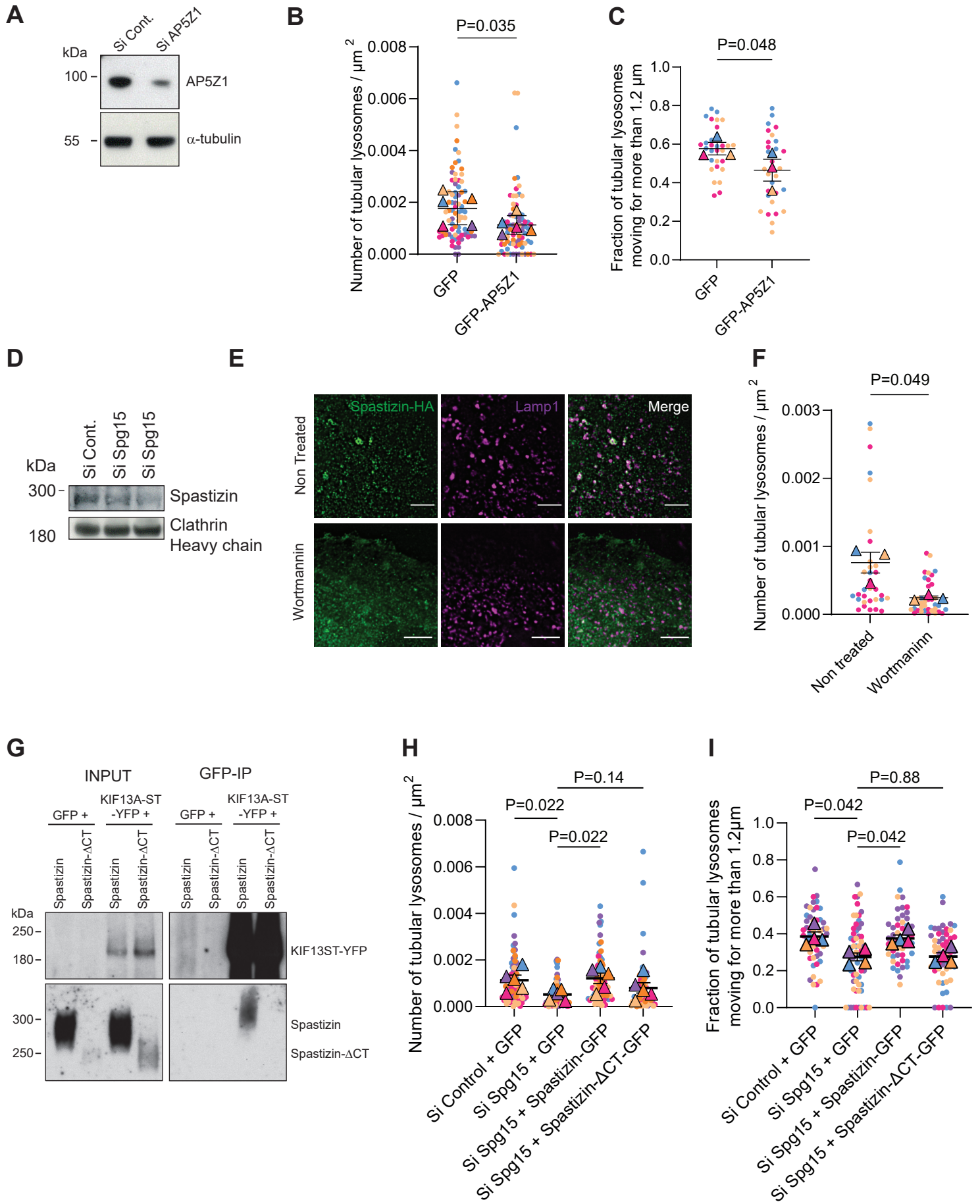

Supplement: S7 Fig — (A) Western blot of wild-type MEFs transfected with a control siRNA or siRNA down-regulating AP5Z1. (B) Quantification of the number of tubular lysosomes in wild-type MEFs transfected with a vector overexpressing GFP-AP5Z1. Superplot: means and SEM, N > 78 cells from 5 independent experiments. Paired t test on the means. (C) Quantification of the proportion of tubular lysosomes moving >1.2 μm over 1 minute in wild-type MEFs transfected with a vector overexpressing GFP-AP5Z1. Superplot: means and SEM, N = 30 cells from 3 independent experiments. Paired t test on the means. (D) Western blot of wild-type MEFs transfected with a control siRNA or siRNA down-regulating Spg15. (E) Images of wild-type MEFs expressing spastizin-GFP and Lamp1-mCherry treated with 100 nM wortmannin for 1 hour. Note the loss of colocalization of spastizin-GFP and Lamp1-mCherry upon wortmannin treatment. Scale bar: 5 μm. (F) Quantification of the number of tubular lysosomes in wild-type MEFs treated with wortmannin. Superplot: means and SEM, N > 32 cells from 3 different independent experiments. Paired t test on the means. (G) Western blots showing co-immunoprecipitation of spastizin-HA, but not spastizin-ΔCT (lacking aa 2,120–2,539) with KIF13A-ST-YFP. Input represents 5% of lysate added to the immunoprecipitation assay. (H) Quantification of the number of tubular lysosomes in wild-type MEFs transfected with siRNA targeting mouse Spg15 (si Spg15) and either GFP, human spastizin-GFP, or human spastizinΔCT-GFP. Superplot: means and SEM, N > 70 cells from 5 independent experiments. RM one-way ANOVA on the means, Holm–Sidak’s multiple comparisons test. (I) Quantification of the proportion of tubular lysosomes moving >1.2 μm over 1 minute in wild-type MEFs with siRNA targeting mouse Spg15 (si Spg15) and either GFP, human spastizin-GFP, or human spastizinΔCT-GFP. Superplot: means and SEM, N >45 cells from 4 independent experiments. RM one-way ANOVA on the means, Holm–Sidak’s multiple comparisons test [file pbio.3002337.s007.pdf]

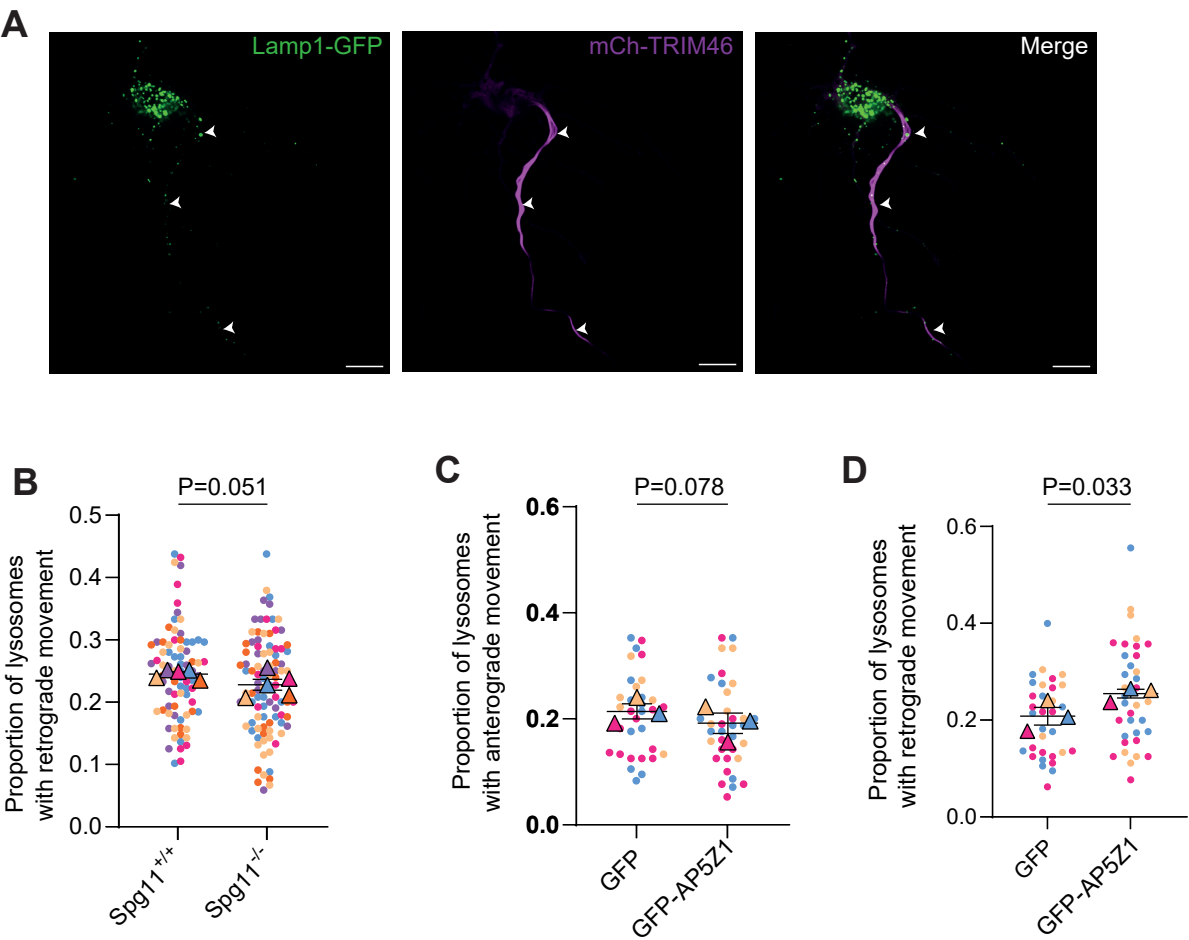

Supplement: S8 Fig — (A) Live image of primary cortical neuron transfected with Lamp1-GFP and mCherry-TRIM46 that labels the axon initial segment, allowing us to identify axons (white arrows). Scale bar: 10 μm. (B) Quantification of the proportion of lysosomes that are moving retrogradely along the axon of Spg11+/+ and Spg11−/− primary mouse neurons. Superplot: means and SEM, N > 79 cells from 5 independent experiments. Paired t test on the means. (C) Quantification of the proportion of lysosomes that are moving anterogradely along the axon of Spg11+/+ primary mouse neurons expressing either GFP or GFP-AP5Z1. Superplot: means and SEM, N > 32 cells from 3 independent experiments. Paired t test on the means. (D) Quantification of the proportion of lysosomes that are moving retrogradely along the axon of Spg11+/+ primary mouse neurons expressing either GFP or GFP-AP5Z1. Superplot: means and SEM, N > 32 cells from 3 independent experiments. Paired t test on the means. The raw data underlying panels B, C, and D can be found in S1 Data file. (PDF) [file pbio.3002337.s008.pdf]
