# Supplementary material for: Spatacsin regulates directionality of lysosome trafficking by promoting the degradation of its partner AP5Z1
Source: PLoS Biol. 2023 Oct 23;21(10):e3002337. doi: 10.1371/journal.pbio.3002337 (PMC10621996; doi:10.1371/journal.pbio.3002337)

Pierga et al, S3 Fig

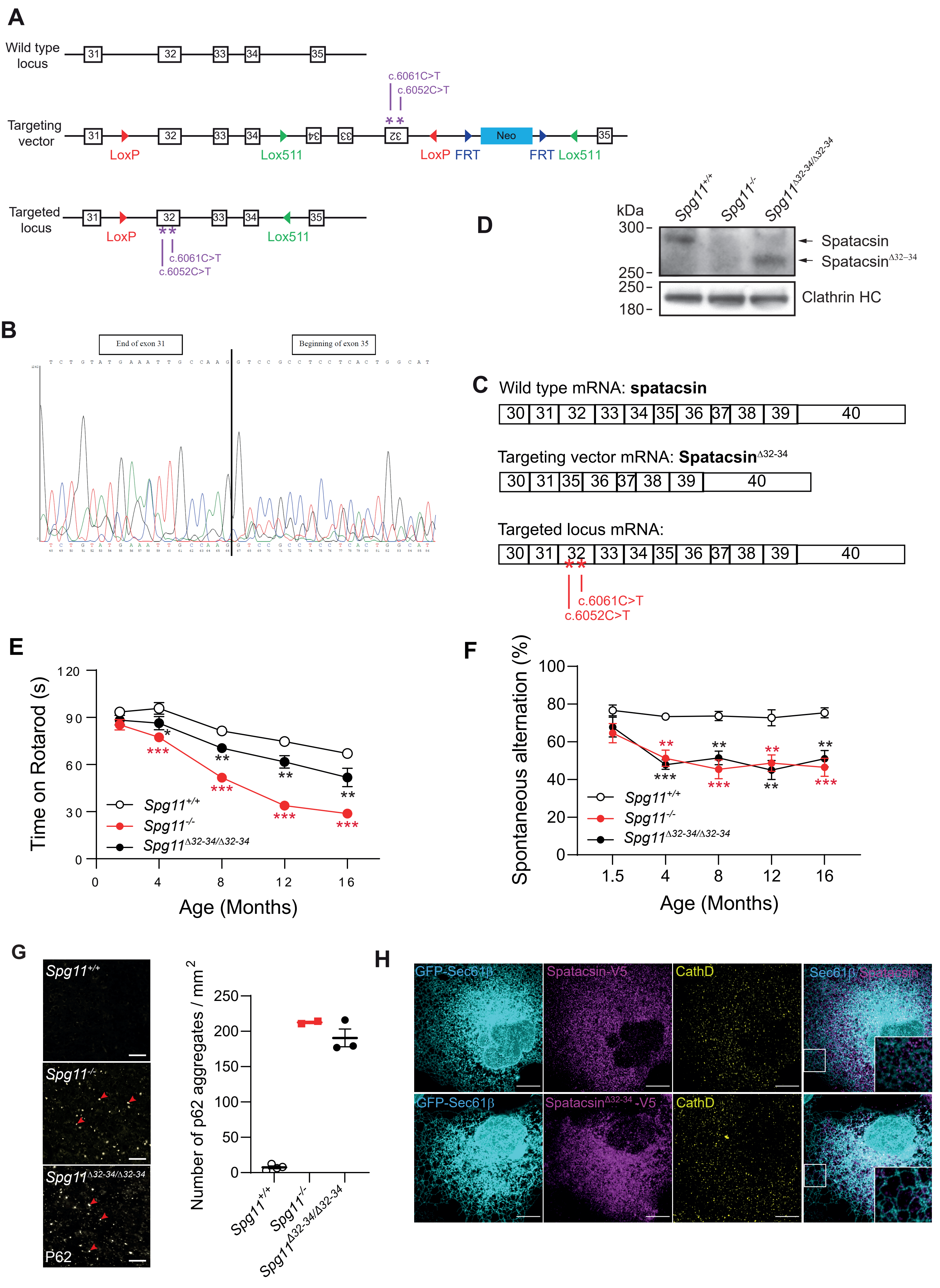

Supplement: S3 Fig — (A) Diagram showing the genomic structure of the mouse Spg11 gene (top), the targeting vector (middle), and the targeted locus upon excision of the neomycin resistance cassette and action of the Cre-recombinase (bottom). Numbers indicate exons. The mutations introduced in exon 32 were c.6052C > T (p.Arg2018*) and c.6061C > T (p.Gln2021*). Scheme adapted from [22]. (B) Sequencing of RT-PCR product obtained from the brains of homozygous mice that incorporated the targeting vector, showing the splicing of exons 32, 33, and 34. (C) Scheme representing the mRNA produced in a wild-type mouse, a mouse that incorporated the targeting vector, or after the action of the Cre recombinase. Note that the intermediate model expressing the floxed allele showed splicing of exons 32 to 34 with conservation of the reading frame between exons 31 and 35. It was thus equivalent to a functional deletion of exons 32 to 34, leading to expression of a protein called Spatacsin∆32–34. (D) Western blot showing expression of truncated spatacsin in Spg11∆32–34/∆32–34 mouse brain. Equal loading was validated by clathrin heavy chain (HC) immunoblotting. (E) The time spent on accelerating rotarod was lower in Spg11−/− and Spg11∆32–34/∆32–34 mice compared to Spg11+/+ mice from 4 months of age. Yet, Spg11∆32–34/∆32–34 mice performance was better than the one of Spg11−/− mice. N = 9 to 15 animals/genotype/age; two-way ANOVA followed by Holm–Sidak post hoc test; *P ≤ 0.05, **P ≤ 0.01 and ***P ≤ 0.001 vs. wild-type (Spg11+/+) mice. (F) Spg11−/− and Spg11∆32–34/∆32–34 mice presented a significantly lower performance from the age of 4 months in the Y-maze test used to evaluate cognitive function. N = 9 to 15 animals/genotype/age; two-way ANOVA followed by Holm–Sidak post hoc test; **P ≤ 0.01 and ***P ≤ 0.001 vs. wild-type (Spg11+/+) mice. (G) P62 immunostaining in cortex of 8-month-old Spg11+/+, Spg11−/−, and Spg11∆32–34/∆32–34 mice. Scale bar: 50 μm. Left: quantification of the number of p62 aggregates pe [file pbio.3002337.s003.pdf]
