# Supplementary material for: Spatacsin regulates directionality of lysosome trafficking by promoting the degradation of its partner AP5Z1
Source: PLoS Biol. 2023 Oct 23;21(10):e3002337. doi: 10.1371/journal.pbio.3002337 (PMC10621996; doi:10.1371/journal.pbio.3002337)
Supplement: S1 Table — Confidence in interaction: A, very high confidence; B, high confidence; C, good confidence; D, moderate confidence. (DOCX) [file pbio.3002337.s012.docx]

| Prey | Accession number | Confidence in interaction | Interaction domain with SPG11 (a.a) |
| --- | --- | --- | --- |
| ALDOA | NM_000034.3 | D | 178-362 |
| ANXA7 | NP_001147.1 | B | 207-336 |
| ARHGEF6 | NP_001293106.1 | D | 1-205 |
| ARIH2 | NP_001304263.1 | D | 136-264 |
| ATXN10 | NM_013236.3 | D | 52-475 |
| BLZF1 | NP_003657.1 | D | 198-362 |
| BTRC | NM_033637.3 | D | 55-363 |
| CARS2 | NP_078813.1 | D | 373-556 |
| CASP2 | NP_116764.2 | D | 338-452 |
| CHN1 | NM_001822.5 | A | 424-459 |
| CHPF | NM_001195731.1 | D | 497-613 |
| CLU | NM_203339.2 | A | 73-198 |
| COG2 | NM_007357.2 | D | 578-738 |
| COPS4 | NM_016129.2 | D | 203-367 |
| DAAM2 | NP_001188356.1 | D | 1-463 |
| DCTN1 var1 | NP_004073.2 | D | 1031-1216 |
| DKFZp434FD46 | AL136884.1 | D | 1-116 |
| DMAP1 | NM_019100.4 | B | 299-427 |
| DNM1 | NP_004399.2 | A | 517-747 |
| EEF1D | NM_001130055.3 | D | 67-254 |
| ENC1 | NM_003633.3 | D | 113-289 |
| EP400 | NP_056224.3 | C | 718-918 |
| EXOC7_var1 | NP_001013861.1 | D | 249-442 |
| FBXO9 | NM_033480.2 | D | 1-155 |
| FRY | NM_023037.2 | B | 1763-1963 |
| GOLPH3 | NM_022130.3 | D | 37-175 |
| HIPK2 var1 | NM_022740.4 | D | 405-569 |
| IFI30 | NP_006323.2 | D | 73-250 |
| IFT172 | NP_056477.1 | D | 750-909 |
| KALRN | NP_001019831.2 | D | 738-1028 |
| KCNaB2 | NM_003636.3 | D | 212-367 |
| KCTD9 | NM_017634.3 | D | 113-389 |
| KDM5D | NM_004653.4 | B | 1-141 |
| LDHA | NM_005566.3 | C | 190-332 |
| MACF1 | NP_036222.3 | B | 938-1321 |
| MAP3K11 | NM_002419.3 | D | 368-501 |
| MCF2L2 | NP_055893.3 | C | 283-365 |
| MKRN3 | NP_005655.1 | D | 415-507 |
| MOAP1 | NM_022151.4 | A | 117-282 |
| MYCBP2 | NM_015057.4 | B | 2185-2578 |
| NEFL | NP_006149.2 | D | 239-446 |
| PBXIP1 | NP_001304664.1 | C | 414-550 |
| PDK2 | NM_002611.4 | D | 23-181 |
| PDS5B | NM_015032.3 | D | 161-473 |
| PIK3CB | NP_006210.1 | D | 465-627 |
| PPP6R2 | NP_001229827.1 | D | 10-138 |
| PSMD2 | NP_001265638.1 | D | 98-244 |
| RALGAPA1 | NM_001346249.1 | D | 2127-2526 |
| RNF31 | NP_001297261.1 | D | 385-517 |
| SMARCE1 | NM_003079.4 | B | 117-247 |
| SMEK2 | NP_001116436.2 | D | 1-244 |
| SPARCL1 | NM_001128310.1 | B | 412-664 |
| SPG7 | NP_003110.1 | D | 610-760 |
| SPTAN1 | NM_001195532.1 | B | 1157-1366 |
| SPTBN1 var1 | NM_003128.2 | D | 1882-1987 |
| SRGAP2 | NP_056141.2 | D | 238-468 |
| TIAM1 | NM_003253.2 | D | 1014-1176 |
| TOMM70A | NM_014820.4 | D | 360-605 |
| TP53BP1 | NM_001141980.1 | C | 1860-1960 |
| TRIP12 | NP_001271145.1 | D | 197-552 |
| TTC8 | NP_653197.2 | B | 1-143 |
| UBR4 | NM_020765.2 | D | 587-729 |
| UPS14 | NM_005151.3 | D | 83-233 |
| UPS8 | NP_005145.3 | D | 673-891 |
| VCPIP1 | NM_025054.4 | D | 744-1017 |
| VPS8 | NM_015303.3 | D | 924-1107 |
| YWHAH | NM_003405.3 | D | 4-210 |
| ZNF821 | NP_001188481.1 | D | 192-412 |
| ZSWIM8 | NP_055852.2 | D | 745-1026 |

**Supplementary Table 1 :** List of preys identified by a yeast two hybrid screen using C-terminal domain of human SPG11 (aa 1943-2443). Confidence in interaction: A very high confidence; B high confidence; C good confidence; D moderate confidence.
