# Supplementary material for: Spatacsin regulates directionality of lysosome trafficking by promoting the degradation of its partner AP5Z1
Source: PLoS Biol. 2023 Oct 23;21(10):e3002337. doi: 10.1371/journal.pbio.3002337 (PMC10621996; doi:10.1371/journal.pbio.3002337)
Supplement: S2 Table — Confidence in interaction: A, very high confidence; B, high confidence; C, good confidence; D, moderate confidence. (DOCX) [file pbio.3002337.s013.docx]

| Prey | Accession number | Confidence in interaction | Interaction domain with SPG11 (a.a) |
| --- | --- | --- | --- |
| ALDH1B1 | NM_025137.3 | D | 30-184 |
| ANKHD1 | NP_060217.1 | B | 144-345 |
| ANKIB1 | NM_019004.1 | D | 66-291 |
| ANKRD17 | NP_056389.1 | D | 137-298 |
| ANKRD35 | NM_001280799.1 | D | 865-911 |
| ANXA6 | NM_001155.4 | D | 409-673 |
| ATP2A2 | NM_170665.3 | D | 2-69 |
| ATP6V0A1 | NM_001130020.1 | D | 17-271 |
| ATXN10 | NM_013236.3 | B | 256-360 |
| BAZ2A | NP_001287834.1 | D | 795-1000 |
| BFSP1 | NP_001186.1 | D | 108-358 |
| BICDL1 | XM_011539000.2 | D | 215-326 |
| BTRC | NM_033637.3 | D | 55-363 |
| c10orf137 | NM_001202438.1 | D | 796-946 |
| c14orf166 | NM_016039.2 | B | 59-233 |
| CAMK1 | NP_003647.1 | D | 166-370 |
| CASP2 | NP_116764.2 | D | 338-452 |
| CCDC14 | NP_001295246.1 | D | 235-420 |
| CFAP46 | NP_001186978.2 | D | 1526-1658 |
| CHN1 | NM_001822.5 | A | 371-459 |
| CHPF | NM_001195731.1 | D | 497-613 |
| CINP | NM_032630.2 | D | 40-177 |
| CLU | NM_203339.2 | C | 35-379 |
| CMYA5 | NP_705838.3 | D | 3566-3721 |
| COG2 | NM_007357.2 | D | 562-738 |
| COQ9 | NM_020312.3 | D | 34-318 |
| CTAGE5 | NM_005930.3 | D | 76-287 |
| DAAM2 | NP_001188356.1 | D | 369-543/  865-947 |
| DBP | NP_001343.2 | D | 251-325 |
| DCTN1 var1 | NP_004073.2 | D | 1020-1163 |
| DCTN2 | NP_006391.1 | D | 173-361 |
| DNM1 | NP_004399.2 | A | 625-721 |
| DNMT1 | NP_001124295.1 | D | 349-538 |
| DRP2 | NP_001930.2 | D | 165-363 |
| DST | NP_899236.1 | A | 4007-4280  4820-4896 |
| EIF3S6 | NM_001568.2 | D | 4-147 |
| EP400 | NP_056224.3 | D | 715-974 |
| FAM184A | NM_001288576.1 | D | 261-397 |
| FBN1 | NM_000138.4 | D | 2224-2505 |
| FBXO9 | NM_033480.2 | B | 1-154 |
| FEZ2 | NM_001042548.1 | D | 159-380 |
| FGFR2 | NP_000132.3 | D | 645-802 |
| FGFR10P2 | NM_001171887.1 | D | 26-215 |
| FRY | NM_023037.2 | D | 1764-1963 |
| FRYL | NM_015030.1 | D | 1694-1906 |
| GAD1 | NM_000817.2 | D | 46-207 |
| GNPTAB | NM_024312.4 | D | 811-1085 |
| GOLPH3 | NM_022130.3 | D | 39-175 |
| HSPC182 | NM_014188.2 | D | 45-185 |
| HUWE1 | NP_113584.3 | B | 1939-2127 |
| IMMT | NM_006839.2 | D | 494-677 |
| KALRN | NP_001019831.2 | D | 724-1092 |
| KIAA1429 | NM_015496.4 | D | 303-657 |
| KIF5C | NM_004522.2 | D | 739-910 |
| KLHL5 | NM_001171654.1 | D | 1-175D |
| MACF1 | NP_036222.3 | B | 919-1364  4805-4891 |
| MADD | NM_003682.3 | D | 1231-1384 |
| MCF2L2 | NP_055893.3 | D | 283-517 |
| MOAP1 | NM_022151.4 | D | 102-282 |
| MPRIP | XP_005256620.1 | D | 1626-1845 |
| MRFAP1 | NP_150638.1 | B | 18-127 |
| MYCBP2 | NM_015057.4 | D | 2185-2578 |
| NAPB | NP_001269947.1 | D | 135-247 |
| NBAS | NP_056993.2 | D | 880-1057 |
| NGFRAP1 | NP_055195.1 | C | 56-111 |
| OTUB | NM_017670.2 | D | 2-216 |
| PBXIP1 | NP_001304664.1 | B | 568-718 |
| PCNT | NM_006031.5 | D | 2448-2573 |
| PDK2 | NM_002611.4 | D | 23-173 |
| PEX5L | NP_057643.1 | D | 469-626 |
| PLA3G12A | NM_030821.4 | D | 47-189 |
| PLXND1 | NM_015103.2 | D | 1379-1497 |
| PMF1-BGLAP | NM_001199662.1 | D | 3-205 |
| PNISR | NM_001322413.1 | D | 164-352 |
| PPP6R2 | NP_001229827.1 | D | 10-138 |
| PTPDC1 | NP_689635.3 | D | 572-782D |
| RABGEF1 var1 | NP_001273989.1 | D | 1-261 |
| RALGAPA1 | NM_001346249.1 | D | 2127-2526 |
| RMND5A | NM_022780.3 | D | 1-240 |
| RNF20 | NP_062538.5 | D | 1-228 |
| RUTBC1 | NM_014853.2 | D | 679-907 |
| RWDD1 | NM_001007464.2 | D | 1-75 |
| SAPS1 | NM_014931.3 | D | 124-257 |
| SCG2 | NP_003460.2 | C | 1-181 |
| SF3B1 | NP_036565.2 | D | 578-900 |
| SMARCE1 | NM_003079.4 | D | 117-297 |
| SMEK2 | NP_001116436.2 | D | 1-244 |
| SPTAN1 | NM_001195532.1 | A | 181-404  1120-1377 |
| SRGAP2 | NP_056141.2 | A | 238-346 |
| TOMM70A | NM_014820.4 | B | 360-593 |
| TRAF3IP1 | NM_015650.2 | D | 563-691 |
| TRIM3 | NM_006458.3 | D | 69-276 |
| TRIM59 | NM_173084.2 | C | 99-236 |
| TTC3var1 | NM_003316.3 | D | 1605-1897 |

**Supplementary Table 2** : List of preys identified by a yeast two hybrid screen using C-terminal domain of human SPG11 lacking domain encoded by exons 32-34. Confidence in interaction: A very high confidence; B high confidence; C good confidence; D moderate confidence.
