# Supplementary material for: Spatacsin regulates directionality of lysosome trafficking by promoting the degradation of its partner AP5Z1
Source: PLoS Biol. 2023 Oct 23;21(10):e3002337. doi: 10.1371/journal.pbio.3002337 (PMC10621996; doi:10.1371/journal.pbio.3002337)

Figure 1A

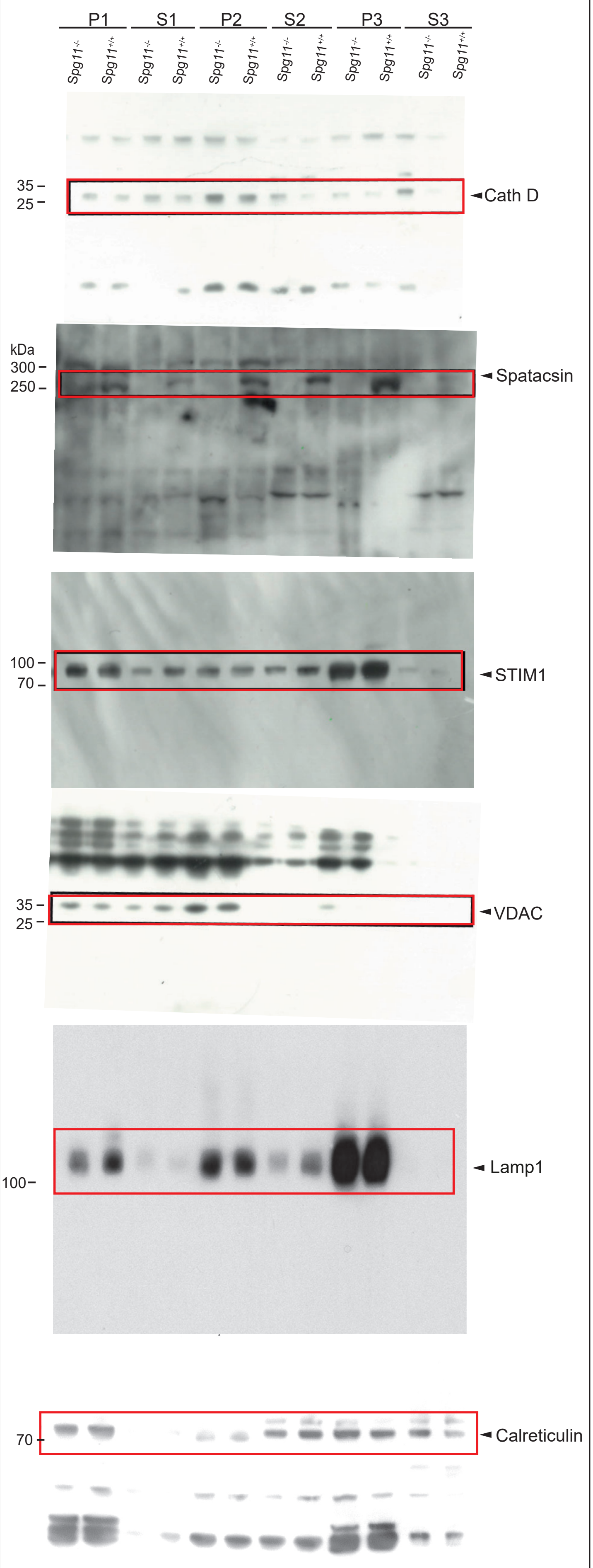

Figure 1B

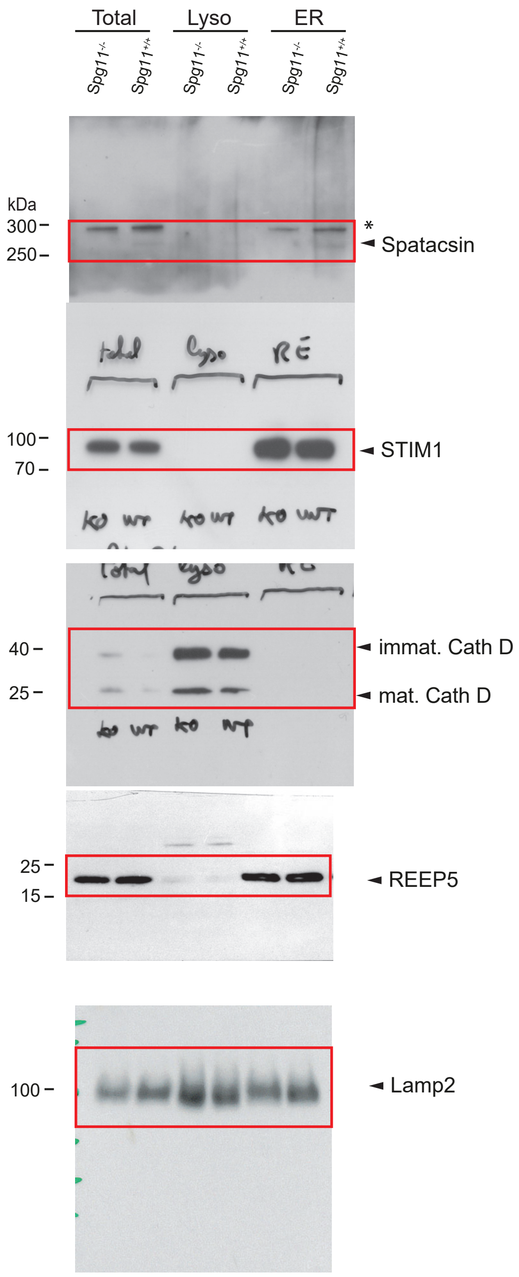

Figure 4A

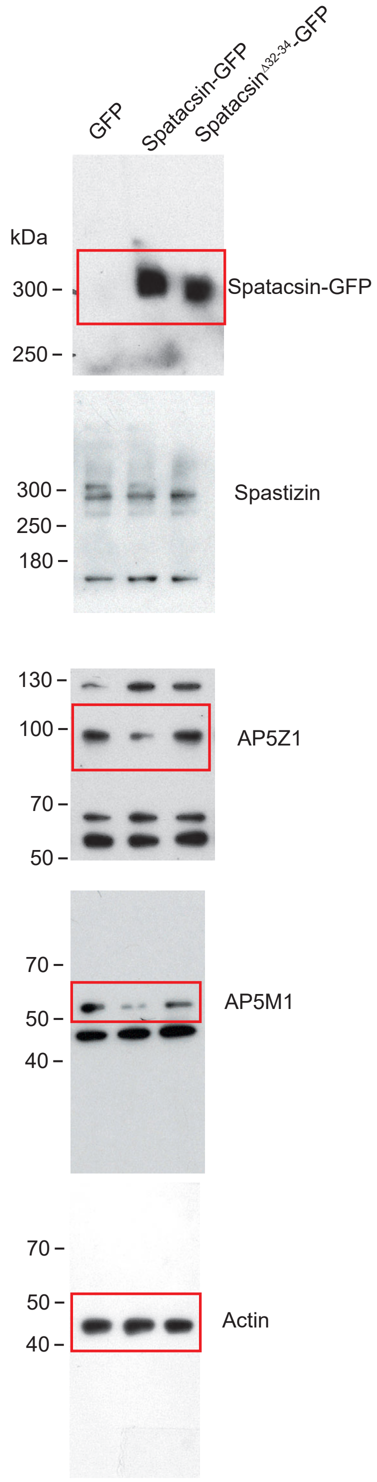

Figure 4B

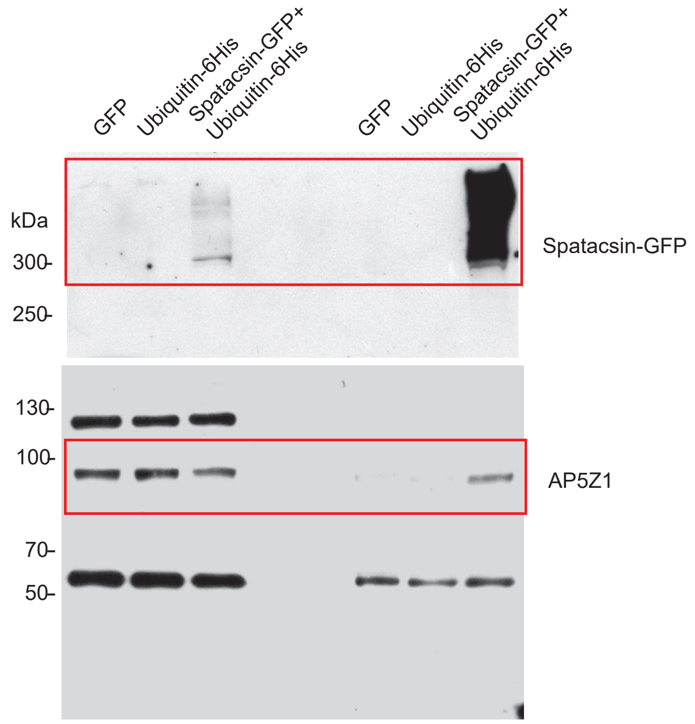

Figure 4C

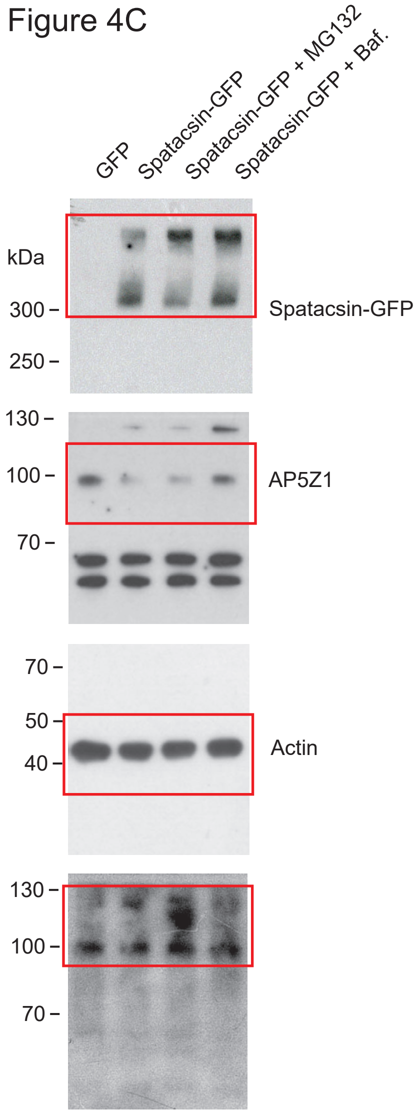

Figure 5A

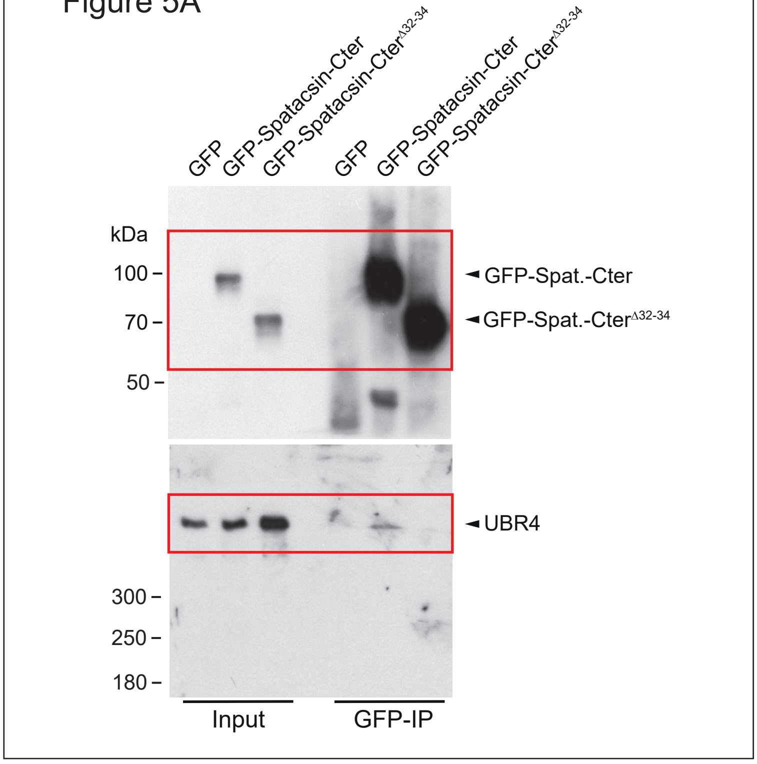

Figure 5B

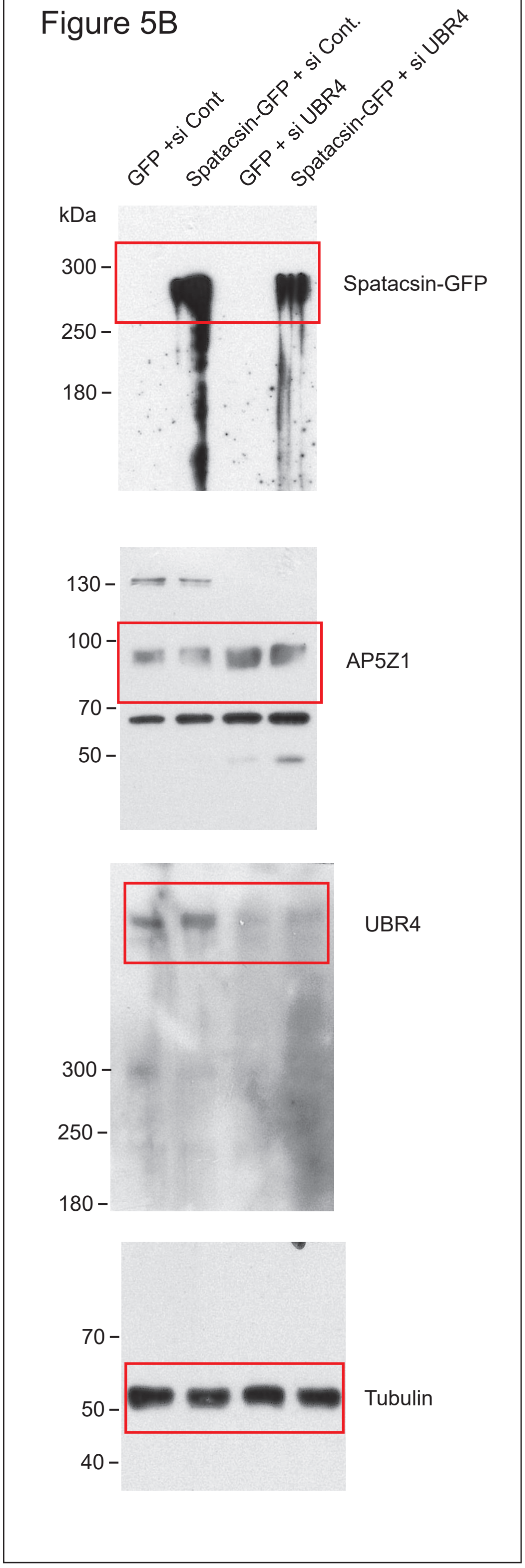

Figure 5C

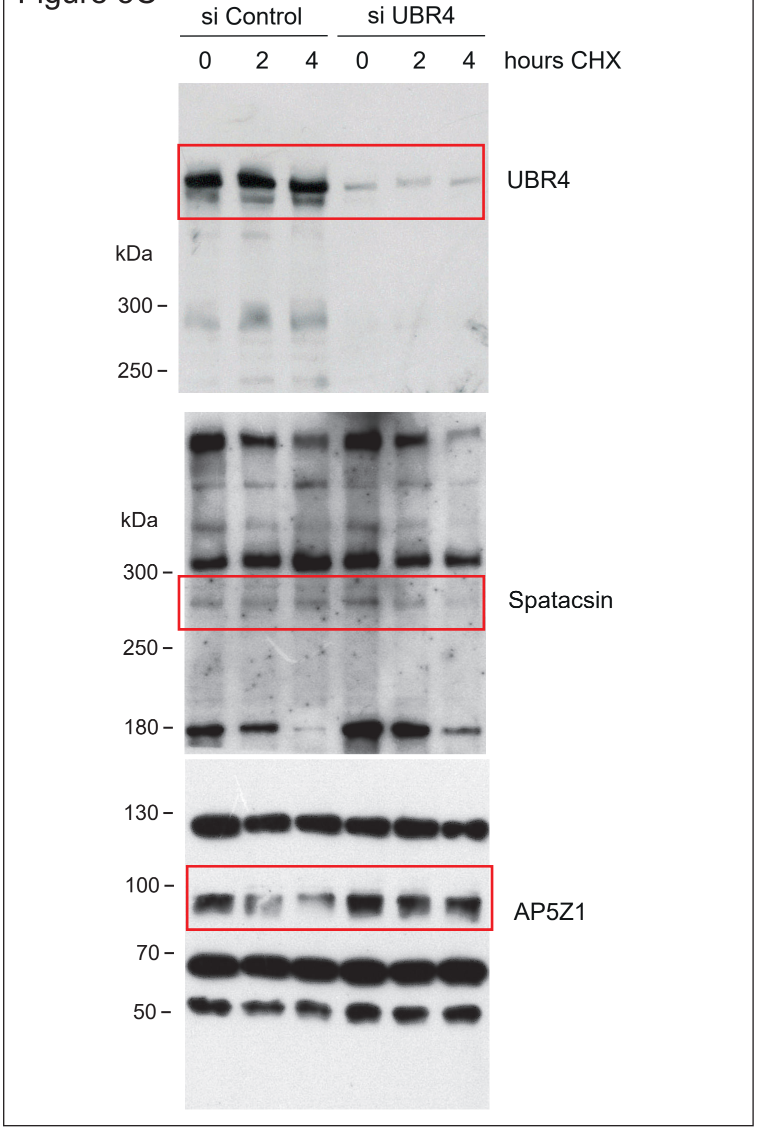

Figure 6D

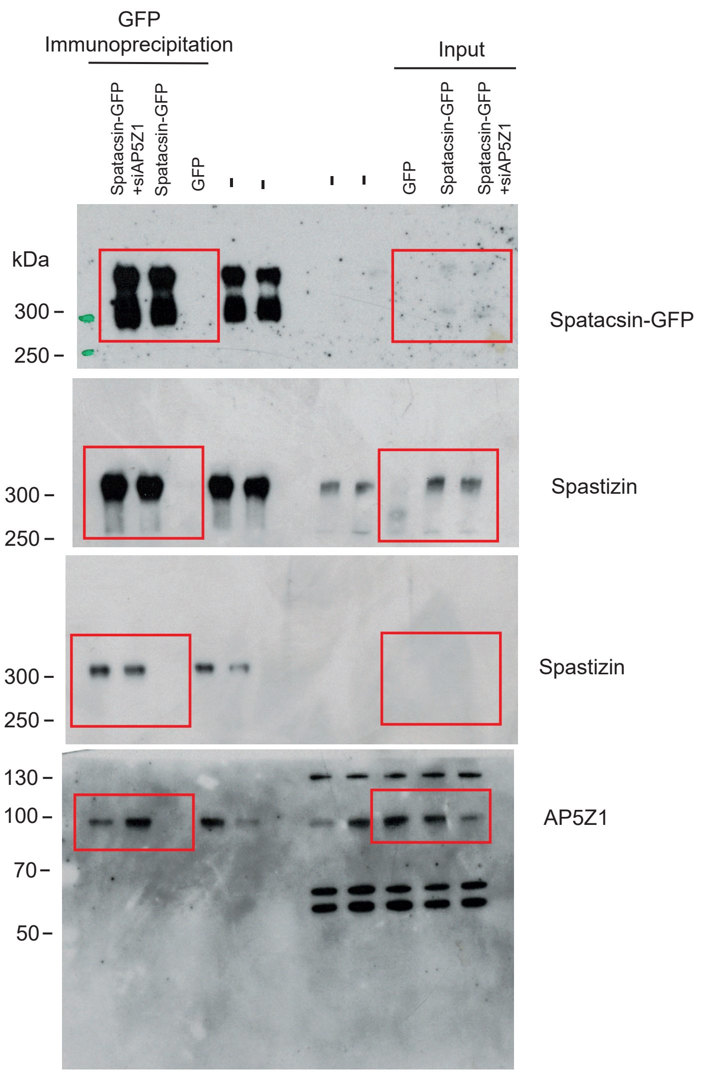

Figure 7G

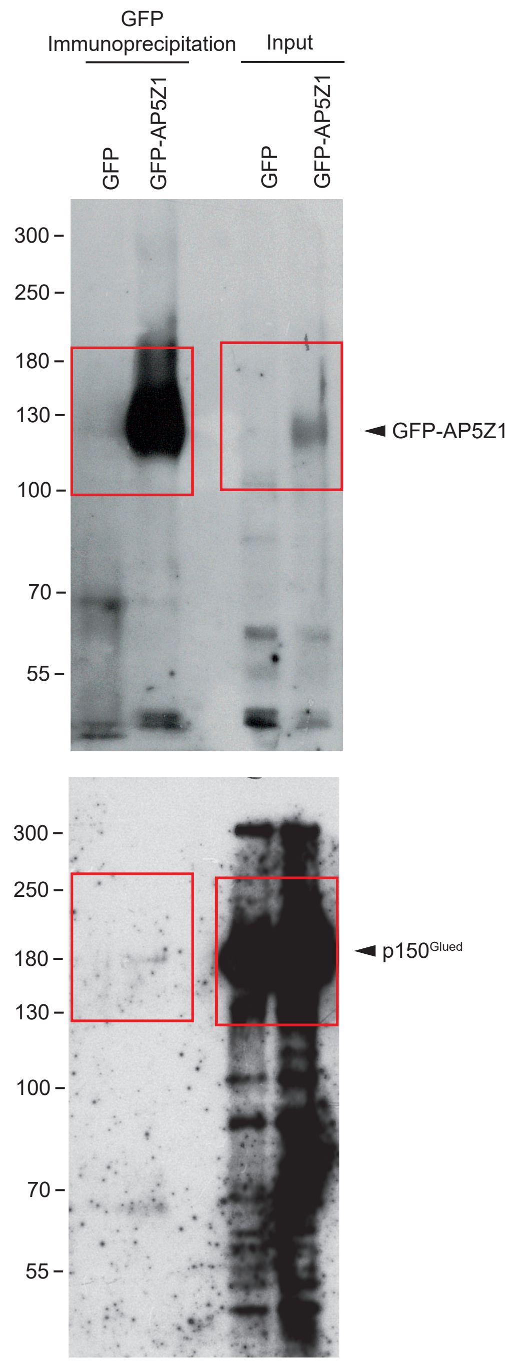

Figure 7D

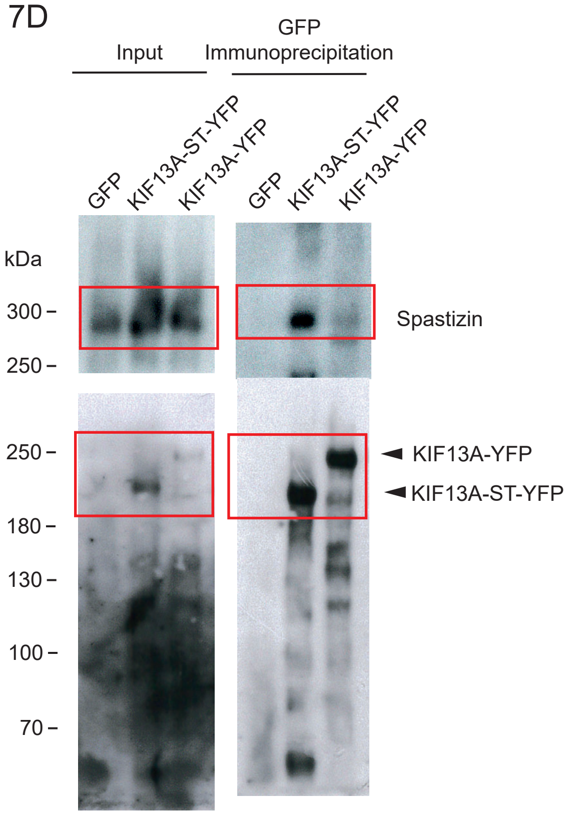

Supplementary Figure 1A

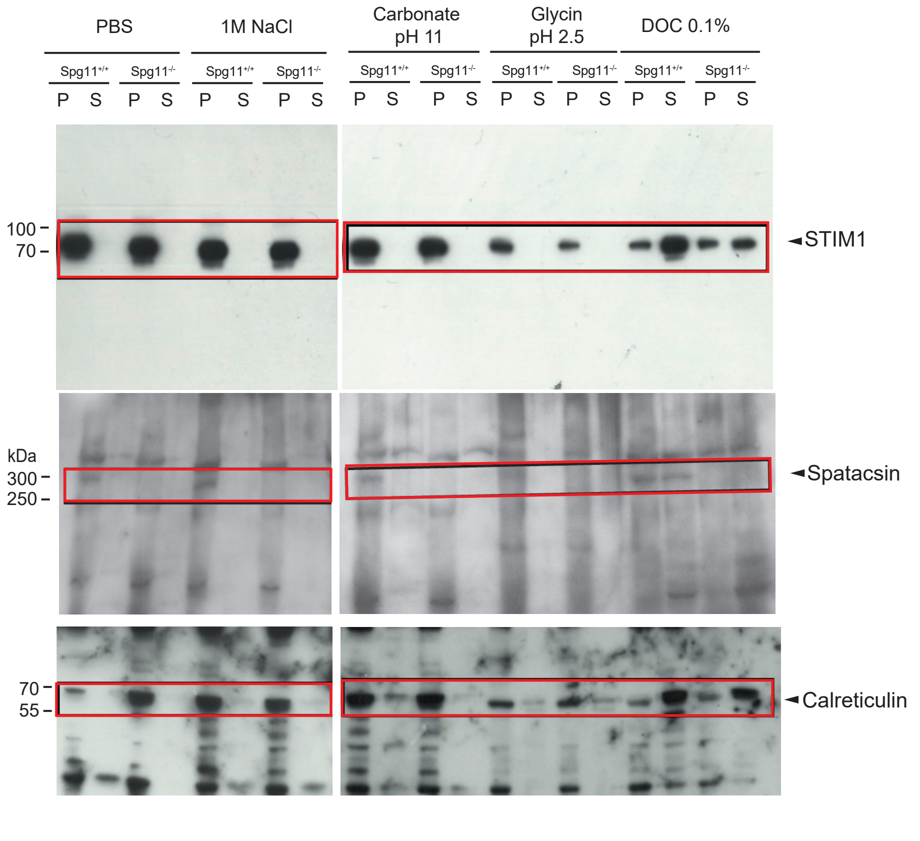

Suppl. Figure 3D

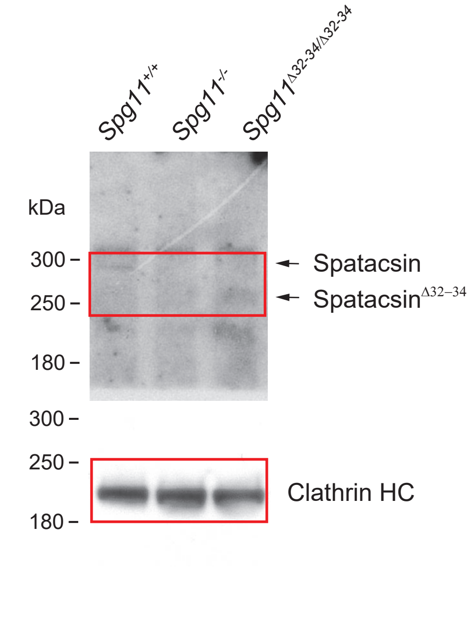

Suppl. Figure 4A

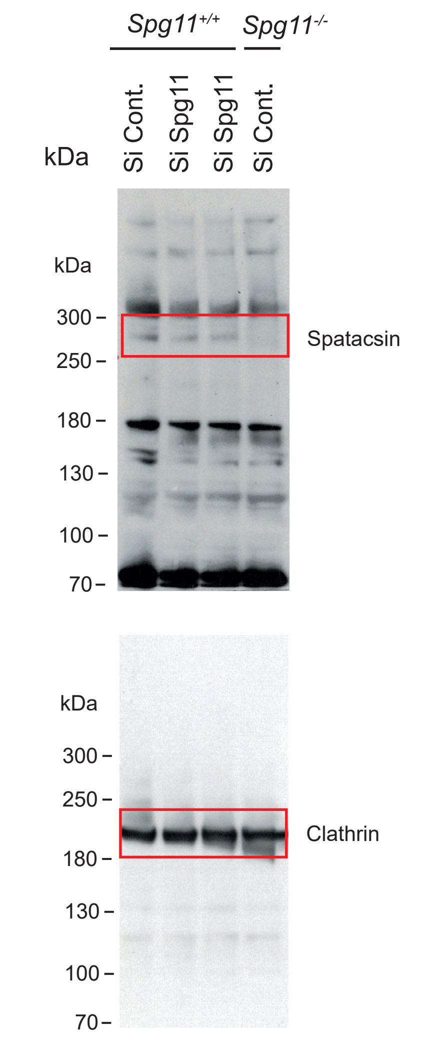

Suppl. Figure 4B

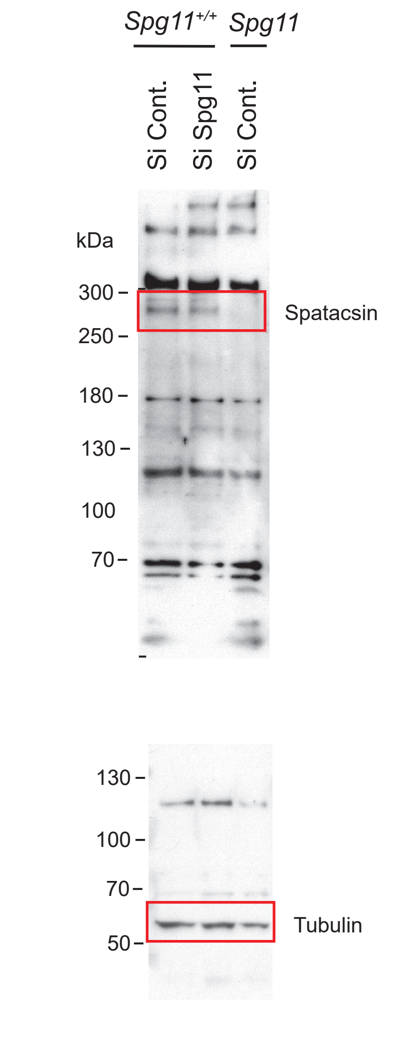

Suppl. Figure 5B

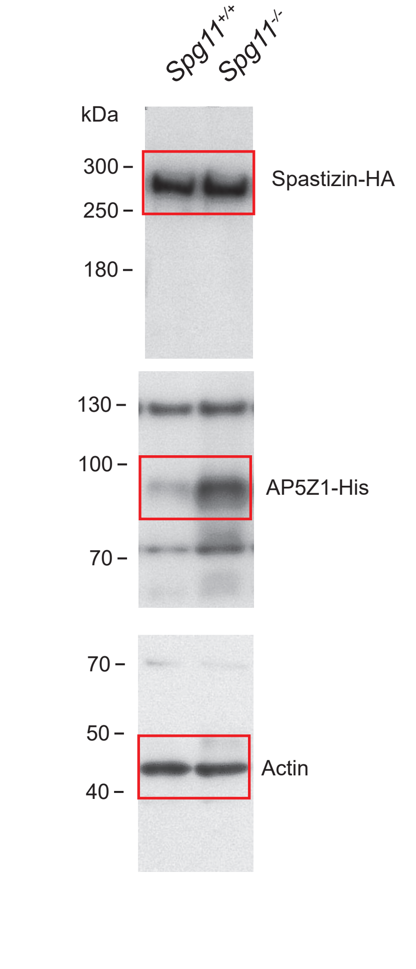

Suppl. Figure 6B

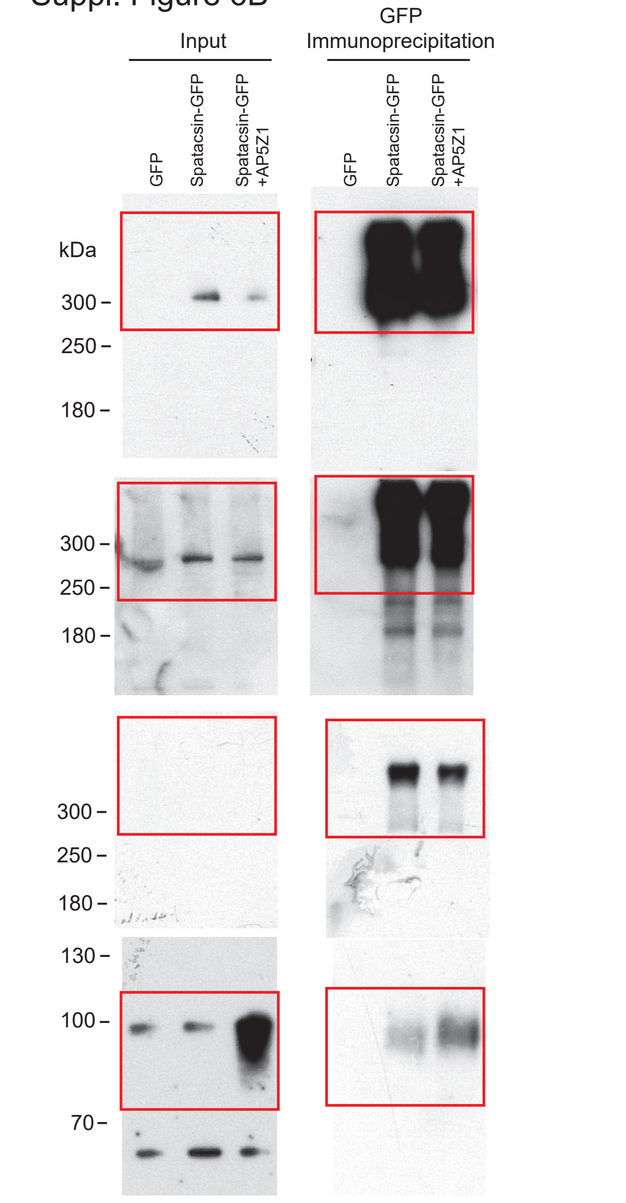

Suppl. Figure 7D

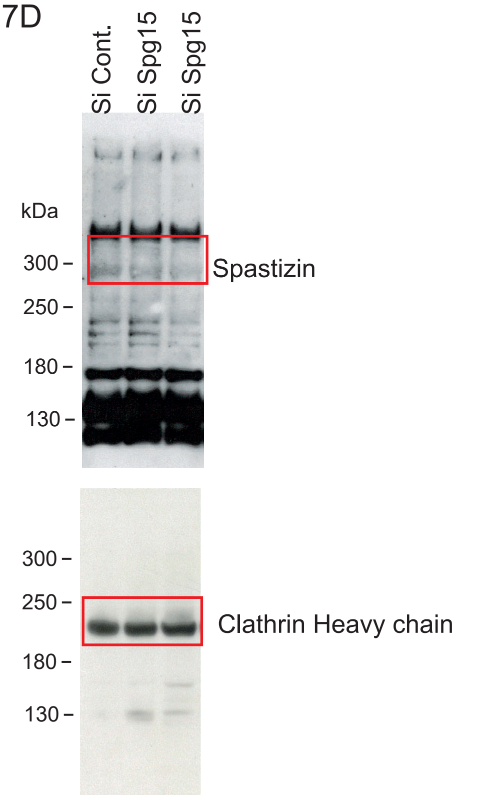

Suppl. Figure 7A

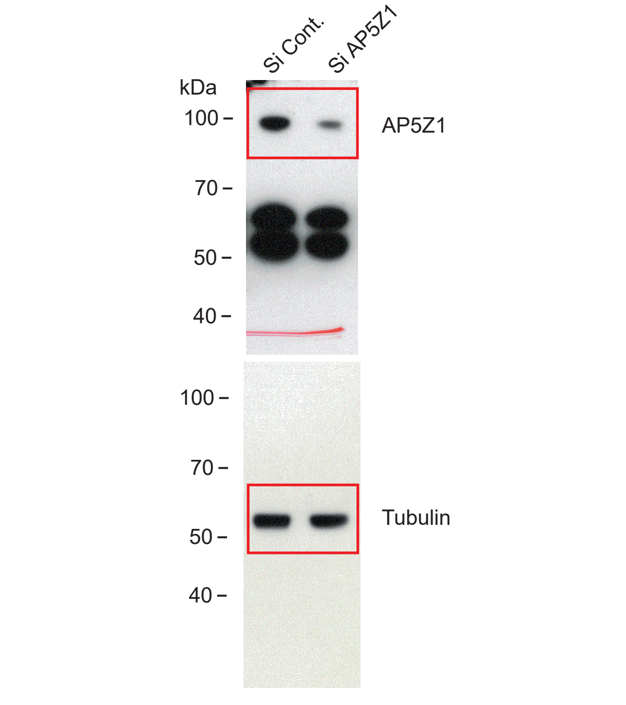

Suppl. Figure 7G

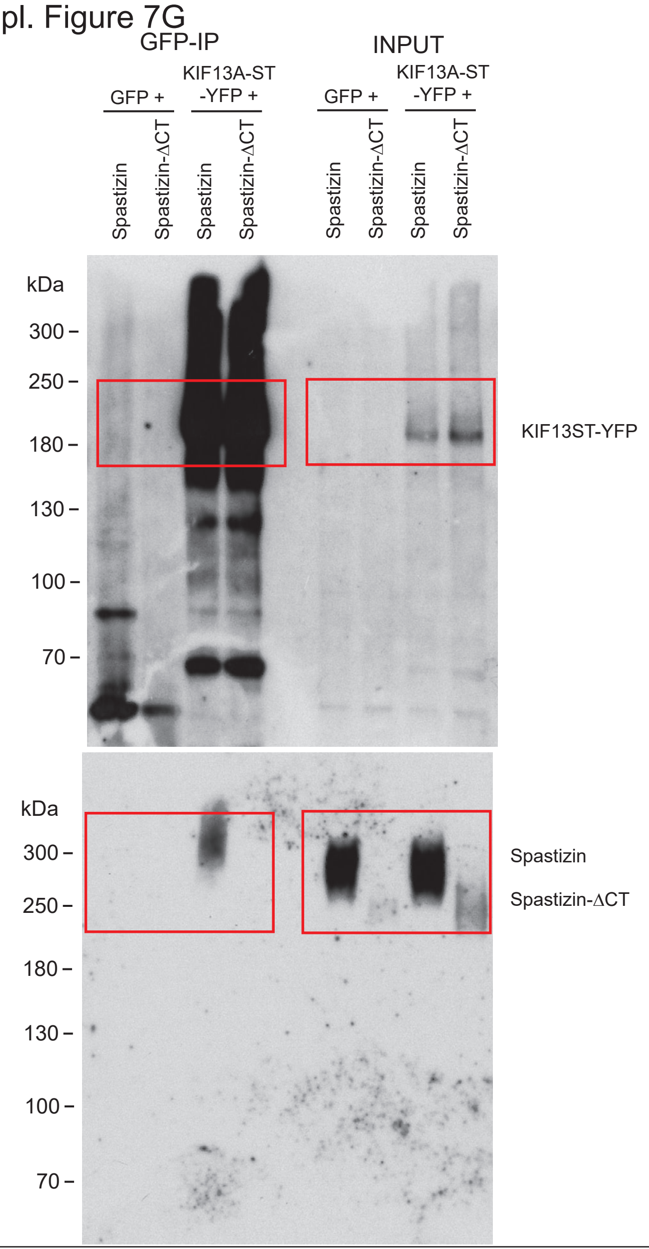

Supplement: S1 Raw Images — (PDF) [file pbio.3002337.s018.pdf]
