# Supplementary material for: Spatacsin regulates directionality of lysosome trafficking by promoting the degradation of its partner AP5Z1
Source: PLoS Biol. 2023 Oct 23;21(10):e3002337. doi: 10.1371/journal.pbio.3002337 (PMC10621996; doi:10.1371/journal.pbio.3002337)
Supplement: Attachment — Submitted filename: Response to reviewers jan 2023.docx [file pbio.3002337.s021.docx]

**Reviewer 1**

This paper identifies a role for the hereditary spastic paraplegia protein spatacsin in lysosome morphology, positioning and dynamics, and undertakes detailed mechanistic studies to try to identify the mechanism for this effect. In doing so the paper elucidates further mechanistic information about the properties of two other hereditary spastic paraplegia proteins, spastizin and AP5Z1. The work is done in mammalian cells and uses a combination of over-expression, depletion and biochemical studies. The main findings are:

1. The authors present evidence that spatacsin is an ER-localised protein.

2. Murine embryonic fibroblasts lacking spatacsin have a reduced number of tubular lysosomes and the remaining lysosomes are less motile. In general, a relationship between tubular lysosome morphology and lysosome motility, often in association with the endoplasmic reticulum (ER), is demonstrated. These tubular lysosomes are catalytically active and acidic.

3. In terms of mechanism of this effect, by combining a yeast-two hybrid and siRNA phenotypic screen, the authors identify a number of spatacsin-interacting proteins that also regulate lysosomal tubulation. The most important of these for the purposes of this paper is UBR4, an E3 ubiquitin ligase.

4. The authors show that spatacsin and UBR4 promote degradation of AP5Z1, and that this property required the ability of spatacsin to interact with UBR4. Somewhat surprisingly, as AP5Z1 is a coat protein, this degradation appeared to occur within the lumen of the lysosome - the authors speculate how this could be in the discussion.

5. The authors then demonstrate that AP5Z1 and spastizin, both hereditary spastic paraplegia proteins, compete for binding with spatacsin.

6. The relationship between spatacsin, spastizin, AP5Z1 and motor proteins in then examined. There is a known interaction between spastizin and KIF13A and expression of a dominant negative KIF13A protein reduced lysosomal tubulation. The authors then demonstrate an interaction between AP5Z1 and the p150Glued dynein/dynactin complex member, then showed that expression of a dominant negative p150Glued protein reduced lysosomal tubulation.

7. Finally, that authors demonstrate the relevance of these findings to neurons, the target cells of hereditary spastic paraplegia, by showing that lysosomal tubulation and axonal transport are reduced in mouse neurons lacking spastacsin, and that depletion of UBR4 or AP5Z1 affected these as expected from the experiments above.

Major comments:

Overall I believe that the key conclusions of this paper are generally convincing and that the work is of high quality. However, I do have some reservations:

1. The localisation of spatacsin on the ER. It is always difficult to be convinced about colocalization of a diffuse punctate marker and the ER. From the STED experiments in figure 1, while it definitely seems that there is some spatacsin on the ER, there also appears to be some spatacsin puncta that are not. I'd like to know if these puncta represent lysosome-associated spatacsin. This is important for interpretation of the subsequent experiments (see point 3 below). I also think quantification of these co-localisation will increase confidence in the results. In addition, a caveat of the immunofluorescence studies is that they use over-expressed spatacsin. I appreciate that there are no good antibodies to endogenous spatacsin, but I don't think this limitation is sufficiently acknowledged. As the claim of ER-localisation is critical for the proposed mechanistic model, and in the absence of experiments with endogenously tagged spatacsin, this makes the biochemical fractionation studies of figure 1C very important. To make these more convincing I would prefer to see additional control markers to verify the separation of lysosomal and ER compartments - e.g. lamp1, lamp2, an ER tubular marker such as a REEP5 or a reticulon.

*Authors response : We agree with the reviewer that the localization of spatacsin is critical, and we appreciate the knowledge of the reviewer concerning the lack of good antibodies to endogenous spatacsin. We better acknowledged this limitation in our revised manuscript (p. 5 and p. 15). We performed extra experiments to convincingly show that spatacsin is indeed localized at the ER. First, we performed 3-color STED experiments to visualize in the same cell spatacsin, the ER and lysosomes (Figure 1D), as well as quantification of colocalization with the ER or lysosomes (Figure 1E). These data indicate that most spatacsin is associated with the ER, and a small proportion of spatacsin colocalizes with lysosomes. Of note, lysosomes that are colocalized with spatacsin are in contact with the ER, supporting the fact that spatacsin is a protein of the ER.*

*Moreover, as requested, we have performed a western blot with Lamp2 and REEP5 antibodies on the ER- and lysosome-enriched fractions (New Figure 1B). This western blot shows that a significant proportion of Lamp2 is present in the ER-enriched fraction, which may be explained by the strong association of ER with late endosomes and lysosomes. Yet the lysosome-enriched fractions that contained no ER markers do not present spatacsin staining, suggesting that spatacsin is either in the ER or in lysosomes associated with the ER that are not positive for cathepsin D. We reformulated the text of Figure 1 according to the new included data (p. 5-6).*

2. The authors generally do a good job of quantifying their results. However, this is lacking for the biochemical experiments (immunoblotting and IP) in figures 4 and 5, and I would prefer to see these quantified (the quantification should include data from repeat experiments so that we can judge the reproducibility of the results).

*Authors response : We agree that our presentation did not indicate that the western blots were repeated several times. We have added quantifications for the western blots present in Figures 4 and 5.*

3. On page 10, referring to the proximity ligation results, the authors comment: "This suggests that the spatacsin-spastizin interaction occurs at contact sites between the ER and lysosomes to allow spastizin recruitment to lysosomes". I'm not sure this statement is fully supported, as mentioned at point 1 above it is possible that some steady state spatacsin is at lysosomes. To fully support this, we'd need to see the PLA signal also convincingly co-localise with an ER marker.

*Authors response : We performed new PLA experiments to show that the spots of spatacsin-spastizin proximity mainly colocalized with the ER marker GFP-Sec61β and the lysosomal marker Lamp1 (new Figure 5C). We also added a quantification of this assay, as requested by Reviewer 3.*

4. In figure 6C and D the effect of spastizin on lysosomal tubulation and dynamics is investigated. Wartmannin treatment is used to do this, as it is known to remove spastizin from lysosomes. However, this is a very indirect manipulation that could have many other consequences and it would be better to demonstrate this directly by showing the effect of depletion of spastizin on lysosomal morphology/dynamics. I also think the role of AP5Z1 in tubulation/dynamics would be better supported with additional experiments to deplete the protein - at present only over-expression is examined.

*Authors response: We added new data to answer this comment. Downregulation of spastizin using siRNA led to lower number of tubular lysosomes and decreased the proportion of dynamic lysosomes, showing that spastizin is required to regulate lysosome motility (Figure 6B-6C Supplementary Figure 7D). We have also added new data regarding downregulation of AP5Z1 (Figure 6A-6C-Supplementary 7A). Both overexpression and downregulation of AP5Z1 using siRNA decreased the number of tubular lysosomes and decreased the proportion of dynamic lysosomes (Figure 6A-6C-Supplementary Figure 6C-D).*

*This observation suggests that the levels of AP5Z1 must be tightly regulated to control lysosome motility. We added discussion about this point as well (p.12-13).*

5. While the experiments showing that over-expression of dominant negative forms of KIF13A and p150Glued affect lysosomal tubulation/dynamics provide good circumstantial evidence that spatacsin influences these lysosomal properties via its interactions with spastizin and AP5Z1 (which bind to these motor proteins), the authors have not shown that the interaction of the motor proteins with spastizin and AP5Z1 is required for this ability to regulate lysosome tubulation/dynamics. This means that the model presented in figure 7 is not fully supported by the data. If the authors have been able to map the binding regions for these interactions then perhaps this could be investigated with rescue experiments, although I appreciate that this is potentially a major piece of work and perhaps outside the scope of this paper. An alternative would be that the authors acknowledged this part of the model as somewhat speculative.

*Authors response : We agree with the reviewer that our data do not show that KIF13A and p150^Glued^ interact directly with spastizin and AP5Z1 to regulate lysosome dynamics. It has previously been shown that the adaptor complex AP2 interacts with p150^Glued^ via the ear domain of AP2 β subunit (Kononenko et al, 2017). It is therefore likely that the interaction of adaptor complex 5 with p150-Glued also occurs via AP5B1 subunit, and thus interaction of AP5Z1 with p150^Glued^ would be indirect. We discussed this point carefully (p.16).*

*Regarding the interaction of Spastizin with KIF13A, it was identified by yeast-two hybrid screen and validated by GST-pulldown (Sagona et al, 2010). This showed that KIF13A interacts with the C-terminal domain of Spastizin, and we discussed this point. To confirm that KIF13A interaction with spastizin is required to promote its role in tubular lysosome formation and dynamics, we performed a “rescue” experiment where we downregulated endogenous mouse spastizin using siRNA and express either full length human spastizin or a human spastizin lacking its C-terminal domain. Unlike full length spastizin, the truncated spastizin did not interact with KIF13A (Supplementary Figure 7G), and did not restore the effect of spastizin downregulation on the number and dynamics of tubular lysosomes (Supplementary Figure 7H-I). These data support the idea that the KIF13A-binding domain of spastizin is required to control lysosome tubulation and dynamics.*

- Are the experiments adequately replicated and statistical analysis adequate?

In general I am not convinced that the statistical tests are applied rigorously in this paper. Most experiments are done three times, but the "n" used for statistical testing is typically chosen as, e.g. the number of cells, number of lysosomes, rather than number of biological repeat experiments. This means that inter-experimental variability is not rigorously taken into account. A more rigorous practice would be to use the mean measures for each of three biological repeats and apply the statistical tests to the three means, so n=3 if three repeats were done. Superplots would be a nice way to graphically display these data.

*Authors response : We agree with the comments of the reviewer regarding data presentation. We have therefore changed the presentation of all graphs of the manuscript using superplots that allow us to show all the points that were analyzed as well as the mean value for each biological replicate, and performed statistical analyses by comparing the biological replicates as proposed in Lord et al, JCB 2020 (*[*10.1083/jcb.202001064*](https://doi.org/10.1083/jcb.202001064)*).*

Minor comments:

1. In supplementary figure 3D I cannot honestly say that I see the smaller band.

*Authors response : We agree that this western blot was not clear. We have provided a new western blot showing expression of a smaller form of spatacsin in Spg11^Δ32-34/Δ32-34^ mouse brain (Supplementary Figure 3D).*

2. When first called out, I expected supplementary tables 1 and 2 to show the list of interactors with wild-type spatacsin and spatacsind32-34 respectively, but this is not what they show.

*Author response : We have added two supplementary data tables (Now Supplementary Tables 1 and 2) to give the list of interactors of wild-type C-terminal domain of spatacsin and spatacsin^Δ32-34^, respectively.*

*Supplementary Tables 3 and 4 now refer to the analysis of the downregulation experiments by respectively the neural network method and the tubular lysosome detection method.*

3. The experiments in Figure 4A are a little problematic in the way that they are called out. The first call refers to just a small subset of the data in the figure, and the figure is then called out at various points later in the paper. This is quite confusing. Is there any way this could be simplified?

*Authors response : We agree with the reviewer that Figure 4A was called at various points of the manuscript. We have modified the presentation of Figure 4 and Figure 5. We have included new Figure 4C to show that downregulation of UBR4 prevents the degradation of AP5Z1 upon overexpression of Spatacsin-GFP, but also in basal conditions in wild-type fibroblasts. The co-IP that was originally presented in Figure 4A has now been moved into Supplementary Figure 6A.*

4. The section on page 10: "Spatacsin also interacts with spastizin, and is required to recruit spastizin to lysosomes (Hirst et al., 2021). ........ We hypothesized that spatacsin interaction with spastizin was required for spastizin localization to lysosomes." Is odd, as the authors seem to be hypothesising an observation that they have just said has already been demonstrated.

*Authors response : We agree that these sentences were odd. We have rephrased the paragraph (p. 11).*

5. Can the authors explain why there is so little interaction between wild-type KIF13A and spastizin?

*Authors response : The interaction domain of spastizin with KIF13A is close to the motor domain according to the two-hybrid data published by Sagona et al (2010). The dominant negative construct of KIF13A that is devoid of the motor domain (KIF13A-ST) may thus facilitate access of spastizin to binding domain. We have commented on this point in the text (p.13).*

6. In figure 6G p150Glued signal is also present in the control IP lane, which casts doubt on the specificity of the interaction. Could the authors generate a cleaner result?

*Authors response : We repeated the co-immunoprecipitation of p150Glued with GFP-AP5Z1, increasing the number of washes. We provide a new western blot showing no P150Glued signal in the control line of the immunoprecipitation (Figure 6G).*

7. I would be interested to see how AP5Z1 expression differs between neurons with and without spatacsin- we would expect similar results to those shown in the MEFS.

*Authors response : We have not checked the levels of AP5Z1 in neurons with and without spatacsin. However, the complete knockout of spatacsin strongly modifies the levels of its partners spastizin and AP5Z1. We previously showed that spastizin levels are decreased by >90% in Spg11 knockout brain (Branchu et al, 2017). Furthermore, the levels of AP5Z1 have been shown to be decreased by ~50% in fibroblasts of SPG11 and SPG15 patients (Hirst et al, 2015). It was proposed that spatacsin may regulate the stability of its partners spastizin and AP5Z1, and it is thus likely that AP5Z1 levels are lower in Spg11 knockout neurons compared to control neurons.*

*In our work, we show that spatacsin promotes the degradation of AP5Z1. Therefore, spatacsin may contribute to tightly regulate the levels of AP5Z1 by contributing both to its stability and to its degradation. It is possible that other degradation mechanism(s) may exist and could explain the lower levels of AP5Z1 in Spg11 knockout cells. We have discussed this point (p.18).*

**Reviewer #2** (Evidence, reproducibility and clarity (Required)):

In this study Pierga et al. report that SPG11 (spatacsin) is an ER-resident protein involved in the regulation of ER-lysosome contact sites (in particular tubular lysosomes) and subsequent faster motility of tubular lysosomes, as well in the degradation of AP5Z1 (SPG48), which forms a heterotrimeric complex with SPG15 (spastizin) and SPG11. This complex has been localized by several groups on the cytoplasmic side of LAMP-1-positive lysosomes. In addition, mutations in SPG11, SPG15, and SPG48 patients share various clinical features and were supported by biochemical/cell biological data from Spg11 and Spg15 KO mouse models and cultured cells both from patients and mice, respectively, demonstrating e.g. accumulation of autolysosome storage material, defects in the autophagic lysosome reformation process, and the loss of cortical motoneurons and Purkinje cells.

Major concerns:

i) Fig. 1, 2, 3: major disadvantage of this study is the analysis of overexpressed proteins (SPG11-V5, GFP-Sec61, and Lamp1-mCherry) which might contribute to the observed strong expression of SPG11-V5 in the ER/ER-enriched fraction. The results should be compared with the endogenous expressed proteins.

*Authors response : As stated by reviewer 1, there are no good antibodies to endogenous spatacsin, and therefore we have to rely on expression of tagged spatacsin to study its localization by immunocytochemistry. For the colocalization with the ER, we stained the latter by GFP-Sec61 that is a widely used marker for this compartment. To confirm our results, we added new STED images with REEP5 antibody to stain the ER, and Lamp1 antibody to label lysosomes, avoiding overexpression of proteins to label the subcellular compartments (Supplementary Figure 1C). Furthermore, as it is not possible to localize endogenous spatacsin by immunostaining, we addressed its localization by biochemical fractionation and western blots comparing wild-type and Spg11 knockout samples (Figure 1A-B).*

*For Figure 2, the data presented were indeed obtained using transfection of Lamp1-mCherry. However, we confirmed our observation of Figure 2A using alternative staining of lysosomes (Lysotracker or loading of lysosomes with Texas-Red Dextran, Supplementary Figure 2A-B). We therefore think that our data presented in Figure 2 are valid, and that the effect we observed on tubular lysosomes was not affected by expression of Lamp1-mCherry.*

*In Figure 3, the lysosomes were labelled with Texas-Red Dextran, and thus all the data presented in figure 3 do not rely on overexpression.*

In Fig. 1C the lack of the mature Cathepsin D form which is proteolytically generated only in lysosomes from the higher molecular mass precursor is misleading and should be related to presence of lysosomal membrane proteins.

*Authors response: As requested, we have added a western blot showing the immature Cathepsin D and performed a western blot to show the lysosomal membrane protein Lamp2 on the ER- and lysosome-enriched fractions (Figure 1B). This western blot shows that a significant proportion of Lamp2 is actually present in the ER-enriched fraction, which may be explained by the strong association of ER with late endosomes and lysosomes previously described (Friedman et al, 2013). Yet the lysosome-enriched fractions that contained no ER markers do not present spatacsin staining, suggesting that spatacsin is either in the ER or in lysosomes associated with the ER. We reformulated the text of Figure 1 according to the new included data (p 5-6). The 3-colours STED experiment (Figure 1 D, Supplementary Figure 1C) confirmed that spatacsin is mainly associated with the ER and a small proportion of spatacsin colocalizes with lysosomes. Of note, lysosomes that are colocalized with spatacsin are in contact with the ER, supporting the fact that spatacsin may be a protein of the ER.*

Fig. 1D: the TEM image shows only a single lysosome and proposed ER contact zones in wt-MEFs without comparison with Spg11 KO MEFs (only in the quantification). Without double immunogold labeling of SPG11 (and their lack on SPG11 KO cell lysosomes) and known ER contact-site proteins this image and the conclusion are insufficient.

*Authors response : We have added an image of a lysosome taken from a knockout fibroblast (Figure 1E). As stated above there are no good antibodies to spatacsin for immunostaining, so it will not be possible to perform double immunogold labelling. This prevents us from claiming that spatacsin is a protein enriched at contact sites between the ER and lysosomes. We therefore modulated our result section and discussion accordingly (p.5-6 and p.15).*

ii) The rationale for the selection of the deleted Spg11 region Δ32-34 is not clear. What are the symptoms of this Spg11 knock-in mouse? A more detailed description of the phenotype is required
Is the phenotype including the accumulation of LC3-positive material similar to the phenotype of the SPG11 KO mouse which has been published by Varga et al.(2015) and Branchu et al. (2017) ? If not, is the new mechanisms reported here not so important?

*Author response : We have added new data (Supplementary Figure 3E-F) showing motor and cognitive impairment in mice expressing truncating spatacsin, although the motor dysfunction is slightly less marked than in Spg11 knockout animals. We also checked for accumulation of autophagy markers. We did not use LC3, but p62 that labels substrates to be degraded by autophagy. We observed accumulation of p62 in Spg11 knockout and in Spg11^Δ32-34/Δ32-34^ mouse neurons (Supplementary Figure 3G). These data support the functional importance of the domain encoded by exons 32 to 34 of Spg11. We commented on this in the text (p.8).*

iii) p8/Fig. 3F/Suppl.Fig.3F- the most important part of the manuscript: what are the parameters of lysosomal staining in images that were used to identify genes important for lysosome tubulation by the neural network?

*Authors response : For screening in Figure 3, lysosomes were stained by loading fibroblasts with Texas-Red Dextran overnight, followed by a wash of at least 4 hours. The neural network was first trained to discriminate between control and Spg11^-/-^ fibroblasts, using any parameters of the lysosomal staining, not necessarily lysosome tubulation. This is a completely unsupervised and unbiased method, but one of its drawbacks is that we do not know which parameters were used by the network to discriminate between control and Spg11^-/-^ fibroblasts. Therefore, we validated the classification performed by the neural network on a data set independent from the training set before using it for the screening. We rephrased the paragraph to make it clearer (p.9).*

I cannot understand how the authors predict the probability of the cell to be considered as an Spg11 KO fibroblast (why not as an Spg11 Δ32-34 knock-in fibroblast?) as the basis for the selection of interaction candidates.

*Author response : The neural network was trained on sets of images obtained from wild-type and Spg11 KO fibroblasts, which were expected to represent extreme phenotypes linked to spatacsin function (presence versus absence of spatacsin). We could therefore predict the probability of cells to be considered as Spg11 KO, not as Spg11 Δ32-34 fibroblasts. We clarified this in the text (p9).*

A simple statement that the neural network approach identified those genes is too weak and requires more convincing experimental data. It has to be shown at least for the 8 positive genes in both approaches how the siRNA treatments of these genes phenocopied the lysosomal changes and of course the effect of the downregulation on the protein level of their products both in wild-type control and Spg11 Δ32-34 knock-in MEF. The Suppl. Fig.3F is completely unclear. How were the Y2H interaction partner validated? Did the authors use the identified 8 interaction candidates as full length bait to demonstrate the interaction with the Spg11 exons 32-34 ?

*Author response : The purpose of the siRNA screen was to quickly identify putative candidates important for the regulation of lysosome dynamics. We identified 8 candidates possibly implicated in lysosomes dynamics based on the two analysis methods. We have added in Supplementary Figure 4 C-D the effect of both siRNA on lysosomal function by the two methods of analysis compared to the effect of siSPG11. However, here we aimed to identify candidates and we do not claim that every one of these eight proteins were indeed implicated in the regulation of lysosome dynamics. We corrected the text, accordingly, stating that the products of the 8 identified genes are good candidates to regulate lysosomal function (p.10). We validated the role of one of the identified candidates, UBR4, and we showed that the UBR4 siRNA indeed downregulates the protein level (Figure 4C). We only validated the interaction of spatacsin Cter with UBR4 by co-immunoprecipitation (Figure 4B).*

*For the 7 remaining candidates, full characterization would indeed be required to validate their role and elucidate their mechanisms of action, but this is out of the scope of this manuscript.*

p8/Fig.3F: the genes identified in both approaches have to be listed in the Fig. 3F-Table.

*Authors response : We have added in new Figure 3F the list of the 8 candidate genes that could contribute to regulate lysosome function.*

The GO process- ubiquitin-dependent protein catabolic process is neither positive for the neural network nor for the directed analysis but positive for both analyses? Please explain. Similarly, the GO process proteolysis involved in cellular protein catabolic process -is not positive for the neural network analysis but again positive for both analyses.

*Authors response : We agree with the reviewer that Table 3F in its older version could be confusing. GO analysis is based on “enrichment” of biological processes within a list of proteins. As we did not have the same number of proteins in the 3 analyses provided in original Table 3F, we got variability in the identified biological processes. To simplify, we have therefore chosen to present only the GO analysis for the 8 candidates that were most likely implicated in lysosomal dynamics according to our two analyses of the siRNA screen which is the most relevant for our study (new Figure 3G).*

For Fig. 3G the mutant ubiquitin-K0 staining in wild-type MEF cells has to be shown as well as for the Spg11 ki/KO MEFs (+ quantification of the respective data)

*Authors response : As stated by Reviewer 4, the expression of lysine-null ubiquitin may impact many different cellular pathways. We therefore removed this part of the data in order to simplify the manuscript (p.10).*

iv) The interpretation of the Y2H-interactome analysis by the authors is hard to follow. They searched with the exon 32-34 cDNA for binding partner, selected 3 degradative GO processes and showed by overexpression of a mutant Ub-K0 plasmid in wild-type MEFs a decreased number of tubular lysosomes, as well as their dynamics (without showing the control data in Spg11 KO or ki-MEFs). Thus, poly-ub of proteins should be in some way responsible for a lysosomal phenotype of Spg11ki MEFs.

Now they went to AP5Z1, the second binding partner of SPG11, which is reduced in its abundance upon overexpression of Spg11-GFP. I would expect to do the respective control experiment to show that in the absence of SPG11 or in the knock-in cells the amount of AP5Z1 has to increase. However, in the studies by the Huebner group by deletion of Spg11 or the other binding partner Spg15, no increase of AP5Z1 protein levels has been observed. The authors have to comment on this discrepancy.

*Authors response : We agree that this is an important point to discuss, and we failed to do it in our first version. Our work shows that overexpression of spatacsin promotes the degradation of AP5Z1. Furthermore, as requested in the point (vi), we overexpressed tagged AP5Z1 and spastizin in wild-type and Spg11 knockout MEFs. We observed significantly higher expression of AP5Z1 in knockout compared to wild-type MEFs, whereas expression of spastizin was similar in both cell types. This suggests that upon overexpression of AP5Z1, endogenous spatacsin may promote degradation of AP5Z1. In contrast, the complete knockout of spatacsin strongly modifies the levels of its partners. We previously showed that spastizin levels are decreased by >90% in Spg11 knockout (Branchu 2017). Furthermore, the levels of AP5Z1 have been shown to be decreased by ~50% in fibroblasts of SPG11 and SPG15 patients (Hirst et al, 2015). It was thus proposed that spatacsin may be required for the stability of its partners spastizin and AP5Z1. It is possible that other degradation mechanism may exist, and could explain the lower levels of AP5Z1 in knockout cells. Therefore spatacsin may contribute to tightly regulate AP5Z1 levels by contributing both to its stability, and to its degradation. We have carefully discussed this point (p.17-18).*

Then the authors found that the selected interaction partner of the exon 32-34 sequence, UBR4, does not bind to the Spg11-GFP construct lacking the domain encoded by exons 32-34 but to the C-terminal domain of Spg11-GFP. Unfortunately, all these IP-experiments were shown as cut and paste figures, preventing the direct comparison between the input and the IP protein amounts (since the information is missing what percentage of the input and the IP has been loaded per lane, the evaluation and significance of these Co-IPs are unclear).

*Authors response : We have added in the Figure legend the fact that the input represents 5% of lysate added to the immunoprecipitation assays. Furthermore, the original western blots are included as supplementary material.*

v) p9: AP5 (Z1) is a cytoplasmic protein and can be localized on the cytoplasmic surface of lysosomes. How should the GFP-mcherry-AP5Z1 protein enter the lumen of lysosomes justifying the quenching of the GFP signal? A positive control has to be included in the experiment shown in Fig. 4E demonstrating the effect of MG132 under identical conditions of a protein substrate for proteasomal degradation.

*Authors response : We agree this is an important control. We added a control showing accumulation of ubiquitin in lysates upon MG132 treatment to show it was indeed effective.*

vi) Fig. 5A: In contrast to GFP-mcherry-AP5Z1, spastizin-GFP is localized at the cytoplasmic surface of lysosomes (co-staining with LAMP1-mcherry) in wild-type MEFs. In regard to the incomplete data commented under "minor points Fig.4/Suppl.Fig.4", I suggest to perform a simple control experiment with overexpressed GFP-spastizin and mCherry-AP5Z1 in wild-type MEFs (at the best also in Spg11 KO MEF) with and without bafA treatment, which will clearly demonstrate whether single components of the trimeric Spg11, spastizin-AP5Z1 complex are degraded independently of each other in lysosomes.

*Authors response : As suggested by the reviewer, we overexpressed tagged version of AP5Z1 and spastizin (AP5Z1-His and spastizin-HA) in wild-type and Spg11 knockout MEFs. We observed significantly higher expression of AP5Z1-His in knockout compared to wild-type MEFs, whereas expression of spastizin-HA was similar in both cell types. This suggests that upon overexpression of spastizin and AP5Z1, endogenous spatacsin may promote degradation of AP5Z1, but not the degradation of spastizin.*

vii) why did the authors neither mention nor discuss the described role of SPG11 in autophago-lysosome reformation (ALR)?

*Authors response : We did not discuss ALR in our first version as we did not investigate autophagic conditions. However, due to the well-described role of spatacsin in ALR, we agree that we have to discuss ALR in our manuscript, and we added a paragraph (p.16).*

Minor points

• Figure 1 A, B, D, and G: ER-lysosome contact sites. The quantification of the co-localization of spatacsin-V5 with the ER marker protein GFP-Sec61b has to be given.

*Authors response : We added the quantification performed on STED images showing localization of Spatacsin together with ER and lysosomal markers (Figure 1E).*

Moreover, the authors analyzed overexpressed tagged-proteins only. The results should be compared with the endogenous proteins.

*Authors response : As stated above, there are no good antibodies to endogenous spatacsin for immunostaining. We added new STED images with antibodies against endogenous Reep5 and Lamp1 to label the ER and lysosomes together with overexpressed spatacsin. Regarding endogenous spatacsin, we could only investigate its localization by subcellular fractionation and western blots comparing wild-type and Spg11 knockout samples. We added biochemical data suggesting that spatacsin is enriched either in the ER or in lysosome membrane associated with the ER. These data have been added in Figure 1 and in text (p.5-6) and we added a paragraph in discussion regarding spatacsin subcellular localization (p.15).*

• p8/Figure 3: what does the 'analysis of trained neural networks' mean?

*Authors response : We did not analyzed the trained neural network, but we used this trained neural network to perform image analysis. We clarified the text (p.9).*

• Figure 4: what happens with the other AP5 subunits?

*Authors response : This is a very interesting question. We added data showing that overexpression of spatacsin-GFP induced the degradation of another AP5 subunit, AP5M1 (Figure 4A, Supplementary Figure 5A). We commented the result in the discussion section (p. 17).*

• Fig.4F/Suppl.Fig4: live images of GFP-mcherry-AP5Z1 + lysotracker staining have to be shown both for wild-type MEFs with and without bafilomycin A treatment(as in Fig.4F), and in Spg11 KO and Ki MEFs +/- bafA.

*Authors response : We have added images of GFP-mCherry AP5Z1 in Spg11^+/+^, Spg11^-/-^, and Spg11^Δ32-34/Δ32-34^ without bafilomycin A treatment (Figure 5E) as well as with bafilomycin A treatment (Supplementary Figure 6D). Of note, with bafilomycin A treatment, it was not possible to perform imaging of lysotracker, as this dye accumulates in lysosomes because of the acidic gradient that is disrupted by bafilomycin A.*

**Reviewer #3** (Evidence, reproducibility and clarity (Required)):

This manuscript highlights an interesting localization of spatacsin in the endoplasmic reticulum (ER)-lysosomes contact sites. In addition, it implicates spatacsin in regulating tubular dynamic lysosomes. Mechanistically, the authors propose that spatacsin interacts with UBR4 to promote the autophagic degradation of its binding partner AP5Z1 at the lysosomes. In turn, this would also regulate the amount of spastizin at the lysosomes, which is known to interact with anterograde motors. The authors further show that AP5Z1 interacts with p150Glued. Thus, the balance between AP5Z1 and spastizin at the lysosomes would determine lysosomal trafficking directionality.

Major Comments

1. Several crucial results of the manuscript are based on quantifications performed on immunofluorescence stainings. Data points in graphs show individual cells or individual lysosomes and the authors apply statistical tests on replicates that cannot be considered biologically independent, since they come from the same experiment or even the same cell. It is recommended to show superplots where both the individual data and the average of each independent experiment is indicated as recommended by Lord et al. (J Cell Biol 2020 219 (6): e202001064.). Statistics should be performed only on independent biological replicates.

*Authors response : We agree with the comments of the reviewer regarding data presentation. We have therefore changed the presentation of all graphs of the manuscript using superplots that allow us to show all the points that were analyzed as well as the mean value for each biological replicate, and performed statistical analyses by comparing the biological replicates as proposed in Lord et al, JCB 2020 (*[*10.1083/jcb.202001064*](https://doi.org/10.1083/jcb.202001064)*).*

2. The authors have used yeast two-hybrid to search for spatacsin interactors. Although in the manuscript they refer to supplementary tables that should show these interactors, the available Tables are confusing and refer to the following downregulation experiments.

*Author response : We have added two supplementary data tables (Now Supplementary Tables 1 and 2) to give the list of interactors of wild-type C-terminal domain of spatacsin and spatacsin^Δ32-34^, respectively.*

*Supplementary Tables 3 and 4 now refer to the analysis of the downregulation experiments by respectively the neural network method and the tubular lysosome detection method.*

An experiment to demonstrate that endogenous UBR4 and spatacsin interact by co-immunoprecipitation would be crucial.

*Authors response : We agree with the reviewer that it would be important to test whether endogenous spatacsin and UBR4 are interacting by co-immunoprecipitation. However, we did not manage to immunoprecipitate either endogenous spatacsin or endogenous UBR4, which prevented us to test the interactions of endogenous proteins by co-immunoprecipitation.*

3. Several important experiments to unravel the mechanistic role of spatacsin (Figure 4 and 5) are performed upon overexpression. This is a major limitation of the study and the authors should address it as much as possible. Western blots and immunoprecipitations are shown that appear to have been performed only once and have no quantification. As an example, in Fig 4A the difference in levels of AP5Z1 upon spatacsin overexpression or UBR4 downregulation are very minor. I would be very careful in drawing big conclusions, without additional repetitions and additional experiments in an endogenous setting.

*Authors response : We agree that a lot of our experiments used overexpression. We have now added to the manuscript new data obtained in MEFs where we downregulated either spastizin or AP5Z1 (Figure 6, Supplementary Figure 6 and 7). They confirm the role of spastizin in the regulation of lysosome dynamics. Furthermore, our new data show that levels of AP5Z1 must be tightly regulated as both overexpression and downregulation of AP5Z1 affects lysosome dynamics (p.12). We also discussed these data carefully (p.17-18 ).*

*Furthermore, we agree that our presentation did not indicate that the western blots were repeated several times. We have now added quantifications for the western blots presented in Figures 4, 5 and Supplementary Figure 6. Furthermore, we have also added the data showing that downregulation of UBR4 led to higher levels of AP5Z1 in control fibroblasts (Figure 4C).*

4. The authors suggest a model by which UBR4 recruited by spatacsin is involved in autophagic degradation of AP5Z1. The data shown do not support this conclusion. First, in Figure 4A downregulation of UBR4 does not increase levels of AP5Z1 above the control in lane 1, but only when spatacsin is overexpressed. The effect of downregulation of UBR4 in wilt-type cells on AP5Z1 should be investigated. Secondly, there is no experiment directly proving that the stability of AP5Z1 depends on UBR4.

*Authors response : We have added new western blots (and quantification) in Figure 4C showing that downregulation of UBR4 increased levels of AP5Z1 in control conditions. The fact that downregulation of UBR4 increased levels of AP5Z1 in control conditions suggests that UBR4 contributes to regulating the levels of AP5Z1. However, we agree with the reviewer that our data do not show whether UBR4 directly promotes the degradation of UBR4. We tried some experiments, but we were unable to show that UBR4 actually directly promotes the degradation of UBR4. We have thus amended the text in the result section (p. 10-11) and we discussed carefully this point (p17).*

5. The authors suggest that the interaction of spatacsin with spastizin or AP5Z1 are in competition. This is an interesting hypothesis, however to conclusively demonstrate this, pull-down experiments in KO cells and not upon extreme overexpression should be performed.

*Authors response : We agree that testing the interaction of spatacsin with its partners in SPG15 KO or AP5Z1 KO fibroblasts would be a very good control of our hypothesis. However, we previously showed that the levels of AP5Z1 are lower in SPG15 KO than in control fibroblasts (Hirst et al, 2015), which introduces a bias in the analysis. We therefore concentrated on AP5Z1. As we have no AP5Z1 knockout fibroblasts easily accessible, we performed downregulation of AP5Z1 using siRNA. Our new data show that decreasing AP5Z1 levels in MEFs increased the interaction between spatacsin and spastizin assessed by co-immunprecipitation. These data support that that the interaction of spatacsin with spastizin or AP5Z1 are in competition and have been added in Supplementary Figure 6C.*

Minor comments

1. In figure 1G and 1H the overlapping area between lysosomes and ER is quantified. Considering that the ER occupies a large portion of the field a 90{degree sign} flipped control for both WT and KO would be important to sort out random colocalization. In this direction, it would be also essential to show that the total amount of lysosomes is not different in WT and KO, especially because in figure 1A the lysosomes in WT and KO seem to be different not just in shape but also in number and size. A different number or size of lysosomes affects this analysis.

*Authors response : We added quantifications in Supplementary Figure 1F showing that 90° flipped controls are indeed not capturing the same proportion of contacts between the ER and lysosomes. We also added quantifications in Supplementary Figure 1D-E showing that the average size of lysosomes and the number of lysosomes per unit area are similar in control and Spg11 KO fibroblasts and mentioned it in the text (p.6). If the lysosomal staining appears different in Spg11 KO fibroblasts it is because lysosomes are clustered around the nucleus, an observation that we reported previously (Boutry et al, 2019).*

2. In the second chapter of the Results, the authors state: "we observed by live imaging a higher number of lysosomes with tubular shape in Spg11+/+ compared to Spg11-/- cells", however the number of elongated lysosomes is quantified per area. Why the number of elongated lysosomes is not quantified over the total amount of lysosomes?

*Authors response : The point raised by the reviewer is a fair point. The purpose of our analysis was to compare the number of lysosomes with tubular shape in control and Spg11 KO cells. As the number of lysosomes per unit area is invariant between control and Spg11 KO cells as shown in new data included in Supplementary Figure 1D, normalization to total number of lysosomes or to cell surface reflects the same difference in phenotype.*

3. The In the fourth chapter of the Results, the authors state:" In wild-type MEFs, mCherry was colocalized with lysosomes. In contrast, GFP that is sensitive to pH was poorly colocalized with lysosomes, suggesting that AP5Z1 was mainly inside the acidic subcellular compartment (Figure 4F)." If the aim of the authors is to shown that AP5Z1 is mainly into the lysosome, the amount AP5Z1-mcherry inside and outside the lysosome need to be compared, with a proper statistical analysis. There is also a lot of GFP signal in the cytosol. Why is that?

*Authors response : We agree with the reviewer, we added a quantification of the proportion of mCherry and GFP in lysosomes upon expression of GFP-mCherry AP5Z1 (Supplementary Figure 5C). This quantification show a >2-fold enrichment of mCherry fluorescence in lysosomes compared to cytosol. Regarding GFP, there is a slight enrichment of its fluorescence in lysosome in WT MEFS, and a stronger enrichment in Spg11 KO MEFs.*

*Regarding the presence GFP-AP5Z1 signal in the cytosol, AP5Z1 has no transmembrane domain and may thus exist as a cytosolic protein. Since GFP is quenched in the acidic environment of lysosomes, the GFP fluorescence of the mCherry-GFP-AP5Z1 protein is outside lysosomes, and it appears partly cytosolic. Of note, there is also some cytosolic mCherry signal that is less visible due to the high level of mCherry fluorescence in lysosomes. We clarified this point with the quantification of the proportion of mCherry signal compared to GFP inside the lysosomes.*

4. construct used in the paper is a C-terminal tagged version of spatacsin. The authors should consider to test an N-terminal tagged construct at least for the localization experiments.

*Authors response : We added an immunostaining image of Spatacsin with an N-terminal tag (Supplementary Figure 1B) and mentioned it in the text (p.5-6). As spatacsin with a C-terminal tag, it presents a diffuse distribution that poorly co-localizes with lysosomes.*

5. Figure 5C: a negative control and the quantification are missing.

*Authors response : We added a negative control for the PLA experiment (new Supplementary Figure 6A). We also added a quantification of the PLA experiment (Figure 5C) showing the proportion of PLA spots colocalized with the ER only, the lysosomes only or both compartment to answer comments of Reviewer 1.*

Reviewer #3 (Significance (Required)):

Since spatacsin, AP5Z1 and spastizin are all implicated in hereditary spastic paraplegia, the data are of potential interest not only for basic cell biology, but also to understand the pathogenesis of the disease. In addition, the manuscript proposes a novel model regulating trafficking of dynamic lysosomes.

**Reviewer #4** (Evidence, reproducibility and clarity (Required)):

Pierga et al. reveal subtle differences in lysosome morphology, ER-contact, and trafficking in the absence of Spatascin. These data are replicated with a truncated Spatascin, presumably a loss of function. Two-hybrid screening of the deleted sequence from this truncation for interactors and then asked whether these hits could phenocopy the lysosome morphology changes. This led to an assertion for a role for ubiquitination in these effects. Rather than these hits the group then investigates previously known Spatascin interactors and reports similar complex but subtle abnormalities via overexpression or knockdown of these. While data show overlapping phenotypes by modulation Spatascin, AP5z1, and Spastizin, the manuscript is confusing, leaps from experiment to experiment, and does not provide novel rigorous mechanisitic insight. It conflates all the discrete lysosomes aspects into a collective to link them. The title is over-stated and not appropriate for the experiments.

The localization of endogenous Spatascin is lacking - over-expression is prone to artifact and the punctate data on the V5 suggests much more work is needed to understand where in the cell it is. It would seem much more work is needed here.

*Authors response : As stated by reviewer 1, there are no good antibody to endogenous spatacsin, and therefore we have to rely on expression of tagged spatacsin to study its localization by immunofluorescence. When performing the images, we avoided the cells with the highest ovexpression of tagged spatacsin to limit artifact due to overexpression. We added new data using STED imaging to get better resolution of spatacsin localization and we performed quantification. Yet, we agree that this is still overexpression. That’s why we included subcellular fractionation data where we can detect endogenous spatacsin by comparing WT and Spg11 knockout samples (Figure 1A-1B). These data confirmed that spatacsin is enriched in the ER or in lysosome fraction tightly associated with the ER.*

Furthermore, the EM data (1E) would suggest the far majority of lysosomes are in contact with ER - these seems uncharacteristic.

*Authors response : The EM data in figure 1E indeed shows that the majority of lysosomes are in contact with the ER, as previously shown by other groups (Friedman et al, 2013, Höglinger et al, 2019).*

The phenotypes analyzed are very subtle, and while statistically significant the biological impact is unclear - in many cases individual lysosomes (or lysosome-ER contacts) are considered as an 'n'. While these results are probed across multiple independent experiments the batch effects and how uniform per cell the events are is unclear.

*Authors response : We agree with the comments of the reviewer regarding data presentation. ‘n’ represented individual cells, but did not actually take into account the variability across experiments. We have therefore changed the presentation of all graphs of the manuscript using superplots that allow us to show all the points that were analyzed as well as the mean value for each biological replicate, and performed statistical analyses by comparing the biological replicates as proposed in Lord et al, JCB 2020 (*[*10.1083/jcb.202001064*](https://doi.org/10.1083/jcb.202001064)*).*

In fig 2H critical data are missing - the effect of Spatascin KO on the transition between these morphologies should be considered as in G. Otherwise the relevance is unclear.

*Authors response : We have added this quantification on Figure 2I. It shows that transition of morphology of lysosomes from round to tubular in Spg11 KO cells is still associated with a change of speed, although the average speed attained is lower compared to conditions where spatacsin is present. This shows that loss of spatacsin does not abolish morphological transition of lysosomes but limit their speed in the tubular shape. We commented on this new data in the text (p.7).*

The impact of over-expressing a lysine-null Ub ( Fig 3) is far too crude and non-specific to have meaning here. It is assumed that the only proteins affected are those of interest. This is consistent with much of the paper where "true-true-and unrelated" is more likely than the presumption of causality.

*Authors response : It is true that the expression of lysine-null ubiquitin is really crude and may impact many different cellular pathways. Furthermore, the results obtained with the lysine-null ubiquitin do not contribute to the rest of the paper. We therefore removed the original Fig3G, H, I and Fig 4B and updated the text accordingly (p.10).*

The blots in Fig4 are a relatively poor quality and not quantified over repetition.

*Authors response : There is not much choice for antibodies able to detect spatacsin and AP5Z1. Yet we have validated their specificity by western blot using knockout cells (spatacsin) (Supplementary Figure 4 A-B) or siRNA (AP5Z1, Supplementary Figure 7A; spastizin, Supplementary Figure 7D). We agree that our presentation did not indicate that the western blots were repeated several times. We have added quantifications for the western blots present in Figures 4, 5 and Supplementary Figure 6. We also changed some illustrative western blots to improve quality.*

Controls are missing and Fig5 suffers from a reliance on over-expression - there is a massive over-expression of AP5Z1 which may be affected the stoichiometry of these overall interactions, but with an n=1 its hard to know and its not clear what these data add. Again, while statistically significant (5E and F) due to the nature of data analysis (every lysosome=n of 1) it is not clear how biologically significant UBR4 siRNA or AP5Z1 over-expression is - as the accumulation of AP5Z1 in these two conditions is orders of magnitude apart - again likely unrelated.

*Authors response : As stated above we have changed the representation of the graphs for all analyses. Each point represents one cell, and we included the mean value for each biological replicate. We have also added quantification of Western blots.*

*Preventing degradation of AP5Z1 by UBR4 siRNA or overexpression of AP5Z1 do not indeed have the same effect on total AP5Z1 but do have a similar effect on the interaction of spatacsin with its partners evaluated by co-immunoprecipitation, as illustrated by the quantifications that we have added. We clarified this in the text (p.12). Furthermore, as requested by reviewer 3, we investigated the effect of AP5Z1 downregulation on the interaction between spatacsin and spastizin assessed by co-immunoprecipitation and quantification. These data demonstrate that downregulation of AP5Z1 favored the interaction of spastizin with spatacsin (Supplementary Figure 6C) and strengthen our conclusions.*

Fig 6 begins to conflate the fact that different lysosome morphologies appear to have different trafficking properties even in WT cells and that many of these targets affect morphology - therefore to conclude a direct effect on trafficking seems inappropriate.

*Authors response : In original Figure 6, we showed that Kif13A-ST and p150CC1 changed the proportion of tubular lysosomes (previous Figure 6 and H), and the data showing that these constructs changed the trafficking of lysosomes were presented in Supplementary Figure 5 B-C. We have now moved the data showing the effect of Kif13A-ST and p150CC1 in the main Figure (Figure 6F and 6I) to facilitate the interpretation of the data. Therefore, expression of Kif13A-ST and p150CC1 do not only affect the morphology of lysosomes, but also impaired their trafficking. We thus do not extrapolate lysosome dynamics from their morphology, we actually quantify lysosome dynamics.*

Fig 7 extends this into polar cells (neurons) but still it is not clear whether form (morphology) dictates function (likelihood of trafficking or directionality.

*Authors response :* We did not only analyzed neurons because they are polarized cells, but because neurons are the main cells affected by neurodegeneration observed in absence of spatacsin (Branchu et al, 2017). We added new data on Figure 7 showing that tubular lysosomes in axons are actually more dynamic than round lysosomes, as observed in fibroblasts. We added these data in Figure 7 and text (p.14).

Investigation of lysosome trafficking in axons also allowed us to investigate the directionality of movement, which is difficult in MEFs. We clarified this point in the text (p.14).

In sum, there is a lot of data that collectively points to a partial localization of Spatascin at Er-lysosome contacts and an influence on morphology and trafficking of lysosomes in the cell, but at the end of the day very new mechanism is brought to light.

*Authors response :* The mechanisms regulating trafficking of lysosomes are far from being fully resolved. Our manuscript shows that spatacsin contributes to this regulation by modulating the degradation of AP5Z1. This in turn regulates the lysosomal association of AP5Z1 and spastizin that interact with motor proteins to control lysosomal dynamics.

Reviewer #4 (Significance (Required)):

This manuscript is directed to the basic cell biology community - involving ER, lysosome, and microtubule dependent trafficking. There are some new analytical tools employed and many co-factors and binding partners of Spatascin considered but frankly too many to adequately and rigorously control for. Because of this the manuscript is very unfocused, hard to follow and makes too many assumptions about shared dynamics ? necessarily arising from shared morphology - lysosomes are highly dynamic and can be affected by virtually any change in intracellular trafficking or protein/membrane transport. This is not appropriately considered.

*Authors response :* We have clarified our manuscript to show that dynamics is not necessarily arising from a tubular morphology. It turns out that lysosomes with a tubular morphology indeed are more dynamic that lysosomes with a round morphology. Importantly, in all our experiments dealing with lysosomal dynamics, we have actually included a quantification of lysosome dynamics using time lapse imaging as detailed in methods (p.24-25).
