# Supplementary material for: Spatacsin regulates directionality of lysosome trafficking by promoting the degradation of its partner AP5Z1
Source: PLoS Biol. 2023 Oct 23;21(10):e3002337. doi: 10.1371/journal.pbio.3002337 (PMC10621996; doi:10.1371/journal.pbio.3002337)
Supplement: Attachment — Submitted filename: Response to reviewers.docx [file pbio.3002337.s022.docx]

Response to reviewers

Rev. 1:

The authors have dealt with my comments in a very comprehensive fashion and this is now a very convincing story that I feel will be a significant advance for the field. I have a few very minor points to be considered:

I can see a faint spatacsin band in figure 1B lysosomal fraction, so the comment about spatacsin not being on degredative lysosomes should be softened - especially as the tubular lysosomes whose movement is controlled by spatacsin seem to be catalytically active (as shown in figure 2).

*Authors response: We softened our comment about the absence of spatacsin in degradative lysosomes, both in the result section (p5) and in the discussion (p16). Instead we stated that spatacsin is poorly associated with degradative lysosomes.*

Figure 1D - please add arrow and arrowheads to all images in the panel (not just two), for better orientation.

*Authors response: We added arrow and arrowheads to all images in the panel.*

In suppl fig 3G the marker labelled should be stated on the micrograph panels.

*Authors response: We indicated on supplementary figure 3G that the staining was performed with anti-P62 antibody.*

For the description of the results in figure 4C it would be more correct to say "partially prevented" the degradation of AP5Z1, as some downregulation still seems to happen.

*Authors response: We agree with the reviewer and we modified the text accordingly (p. 11).*

Figure 7H and I should be swapped around so that they can be called from the text in the correct order.

*Authors response: We modified the text (p 15) so that new figures 8H and 8I are called in the correct order.*

These comments are so trivial that I do not feel that I would need to see another revised version. I would like to congratulate the authors on their interesting work, which has been conducted and presented in a thorough and comprehensive way.

Rev. 3:

The authors have put a lot of efforts to answer to the previous concerns. They have also down-played some of the previous conclusions. The results on the lysosomal phenotypes are convincing. In general, I think that the manuscript has improved. However, I still think that the connection between spatacsin and UBR4 and the role in the degradation of AP5Z1 is weak and not convincing (see point 1). If spatacsin has a role in lysosomal tubulation, this can affect AP5Z1 stability independently from a molecular interaction with UBR4.

1) I am still not convinced about the role of UBR4, as proposed by the authors. UBR4 as the other putative interactors with a role in lysosomal tubulation have been identified by yeast-two hybrid, a technique that forces the cytosolic spatacsin protein to the nucleus. It is possible that this leads to increased degradation, owing to the unphysiological localization, and enriches "interactors" linked to this pathway (there is also a proteasomal subunit). In fact, in the screen, UBR4 appears with a moderate score (D). Obviously, these "interactors" would lead to a lysosomal phenotype when depleted. The results in Figure 4 are still unconvincing. Despite the quantification, I cannot see any difference in control condition in Fig. 4C upon UBR4 downregulation. The authors can directly test if downregulation of UBR4 leads to increase stability of spatacsin in a CHX chase experiment This experiment can even be performed on the endogenous proteins. Is AP5Z1 more ubiquitinated when

spatacsin is overexpressed? The IP showing the interaction of spatacsin and UBR4 is also problematic, since the input is not loaded on the same gel, so we cannot judge how efficient is this IP and the control still shows the interaction.

*Authors response: We thank the reviewer for his comments and performed the extra experiments he recommended, which led us to reconsider the mechanisms of AP5Z1 degradation.*

*First, we repeated again the experiment showing that AP5Z1 degradation mediated by spatacsin-GFP overexpression is partially prevented upon UBR4 downregulation. We changed the western blots used for illustration, and included the new experiment in the quantification (N=6). These data are now in new Figure 5B. Then following the reviewer comments, we reconsidered our hypothesis regarding the role of UBR4 in the degradation of AP5Z1. The second point of this review pointed to us the lower levels of spatacsin-GFP upon downregulation of UBR4, an observation that we had previously overlooked and that was actually present in all our experiments. We therefore added quantification of the levels of spatacsin-GFP upon UBR4 downregulation (new figure 5B). This raised the possibility that UBR4 may affect AP5Z1 and Spatacsin. To test this hypothesis, we performed CHX chase experiments as suggested by the reviewer. Downregulation of UBR4 significantly reduced the stability of endogenous spatacsin, and moderately increased the stability of endogenous AP5Z1 (new Figure 5C), suggesting that UBR4 is important for spatacsin stability, and its downregulation likely prevents the degradation of AP5Z1 mediated by spatacsin, leading to changes in relative levels of both proteins. We modified the text in the result section (p 11-12) and in the discussion (p 19) accordingly.*

*We also tested ubiquitination of AP5Z1, and observed that overexpression of spatacsin-GFP promoted ubiquitination of AP5Z1 (new Figure 4B, text p 10), suggesting that such ubiquitination may contribute to its degradation. This is also discussed (p19).*

*As requested, we also provided another WB for the co-immunoprecipitation of spatacsin and UBR4 (new Figure 5A).*

2) Figure 5D. Same as above. In addition, less spatacsin-GFP is immunoprecipitated in the condition of downregulation of UBR4, so it is expected that also less spastizin is pulled-down. Also, why are the bands in the input and IP so different for spatacsin and spastizin?

*Authors response: The new data regarding AP5Z1 and spatacsin stability that we included highlight that downregulation of UBR4 modifies the levels of spatacsin and AP5Z1. We removed the old figure 5D, as the downregulation of UBR4 indeed lowers expression of spatacsin, which introduce a confounding factor in the analysis. We still commented on the difference in bands for spatacisn and spastizin in the input and co-IP. In these experiments, in spatacsin and spastizin systematically looked different in input and co-immunoprecipitation, likely due to the high amount of both proteins in immunoprecipitates. Longer exposure of the input would likely reveal a similar pattern in input, as seen for example in new Figure 4C for spatacsin.*

3) The superplots show now quite clearly that some effects are barely significant (only paired t-test). Of course, this does not mean that these data are not real, but the authors should be aware that there is a certain risk of overinterpretation. The data may become more or less solid with additional experiments. Still, this is a honest way of showing the data. I would recommend to directly show the real p value on the plot.

*Authors response: As suggested by the reviewer, we indicated actual P values on the plots on figures, except in few panels where it would have been hard to read (Supplementary figure 3E and F for example). Furthermore, we toned down some of our conclusions to take into consideration the fact that some P values are very close from the significance threshold (P=0.05).*

Rev. 4:

The authors have done an earnest job of reformatting the data as suggested by other reviewers, plotting individual experiment averages and re-analyzing the data which have added to the strength of the findings. The quality of some blots has also improved.

However, the revised manuscript still falls short of convincing mechanistic insight and new data were not sufficiently provided to address these concerns across the reviewers. Clearly, the loss of spatascin results in less numerous tubular lysosomes - this is seen nicely throughout the paper. But the mechanisms behind spatascin doing this are less well defined. Many details are over-interpreted. Spatascin is found in ER, but is not enriched as the authors claimed - there is lots of spatascin elsewhere in the cell (imaging) and the fractionation of spatascin does not follow STIM1 enrichment, for example. It is localized to ER but the incongruent data for this model is a bit overlooked. UBR4 is a cytosolic E3 ligase, but they authors argue with limited data that it mediates degradation of AP5Z1 (sensitive to bafilomycin, again not well explained) where compensatory/transcriptional effects are not considered (mRNA) and the degradation evidence is incomplete. Since morphology alone (round vs tubular) seems to affect trafficking, it is not entirely clear that the trafficking effects are in addition to morphologic effects (additive) or merely downstream. Likewise, the motor protein interaction and trafficking data are not sufficient to support the robust conclusions.

Rather than focus on a subset of protein-protein interactions and precise mechanistic principles, the manuscript seeks to conclude, in large sweeping statements, the function and inter-molecular interactions of about a half a dozen proteins and falls slightly short throughout. The mechanisms of affecting lysosome morphology, protein recognition and degradation, and motor-protein association are each limited.

*Authors response: As requested by the reviewer, we have toned down our conclusions to avoid over-interpretation:*

- *We do no more claim that spatacsin is enriched in the endoplasmic reticulum. Throughout the text, we indicated that spatacsin is present in the ER (pp 5-6, 16 and 19).*
- *Regarding the function of UBR4, we performed extra experiments as suggested by reviewer 3. We monitored the stability of spatacsin and AP5Z1 following inhibition of translation by a cycloheximide treatment. This experiment showed that upon UBR4 downregulation, spatacsin was significantly less stable, and AP5Z1 moderately more stable. Our new data therefore suggest that UBR4 contributes to stabilize spatacsin. We have added these data (new Figure 5C and text p 11-12) and we modified the discussion accordingly (p19).*
- *We reformulated the description of the role of KIF13A and p150Glued to avoid over-interpretation. We have also clarified the link between trafficking and morphologic effect on lysosomes (p13-14 and p 17). Our data show that both spastizin and AP5Z1 are required for the formation of tubular lysosomes, and both of them also regulate their trafficking. We propose that these proteins perform both functions by interacting with motor proteins. It is therefore possible that the motor proteins that associate with lysosomes to promote their tubular shape may also contribute to their trafficking, which would explain why tubular lysosome are overall more dynamic (p17). The higher traffic observed for tubular lysosomes may thus be a downstream consequence of the recruitment of motor proteins to these lysosomes. We modified the text in the result section (p14) as well as in discussion (p17).*
