# Supplementary material for: Spatacsin regulates directionality of lysosome trafficking by promoting the degradation of its partner AP5Z1
Source: PLoS Biol. 2023 Oct 23;21(10):e3002337. doi: 10.1371/journal.pbio.3002337 (PMC10621996; doi:10.1371/journal.pbio.3002337)
Supplement: Attachment — Submitted filename: 230912 responses to reviewers.docx [file pbio.3002337.s023.docx]

Responses to reviewers:

No reviewer comments.

Changes made to the manuscript:

- As requested, we changed the title of the manuscript, which is now: “Spatacsin regulates directionality of lysosome trafficking by promoting the degradation of its partner AP5Z1”. We agree that this new title is more accurate than the previous one.
- As required by the editor, we indicated in the abstract that our study was performed using mouse samples (mouse embryonic fibroblasts and cultured mouse cortical neurons).
- We indicated in the legend of each figure that the data underlying each graph can be found in the S1_Data file that has been renamed.
- Finally, we have asked the Intact database to make the data we deposited (IM-29701) publicly available. The data will be publicly available by the end of the week (Setember 15^th^) or early next week (September 18^th^).
